# Mitogenomic Analysis of Glirids (Gliridae) and Squirrels (Sciuridae) From Türkiye: Evolutionary and Taxonomic Implications Within the Suborder Sciuromorpha

**DOI:** 10.1002/ece3.70956

**Published:** 2025-02-12

**Authors:** Osman İbiş, Ahmet Yesari Selçuk, Saffet Teber, Mehmet Baran, Klaus‐Peter Koepfli, Haluk Kefelioğlu, Coşkun Tez

**Affiliations:** ^1^ Department of Agricultural Biotechnology, Faculty of Agriculture Erciyes University Kayseri Turkey; ^2^ Genome and Stem Cell Center, GENKOK Erciyes University Kayseri Türkiye; ^3^ Vectors and Vector‐Born Diseases Research and Implementation Center Erciyes University Kayseri Türkiye; ^4^ Department of Forestry, Artvin Vocational School Artvin Çoruh University Artvin Türkiye; ^5^ Smithsonian‐Mason School of Conservation George Mason University Front Royal Virginia USA; ^6^ Center for Species Survival Smithsonian's National Zoo and Conservation Biology Institute Front Royal Virginia USA; ^7^ Department of Biology, Faculty of Science and Letters Ondokuz Mayıs University Samsun Türkiye; ^8^ Department of Biology, Faculty of Sciences Erciyes University Kayseri Türkiye

**Keywords:** *CYTB*, *Dryomys*, Gliridae, mitogenome, phylogeny, Sciuridae

## Abstract

Gliridae and Sciuridae, the most impressive mammalian radiations within the suborder Sciuromorpha, encompass a total of 327 extant species. This study aimed to: (i) characterize the mitogenomes of three sciurid ( *Spermophilus citellus* , *Spermophilus taurensis* , and *Spermophilus xanthoprymnus* ) and three glirid ( *Glis glis* , *Dryomys nitedula* , and *Dryomys laniger* ) species from Türkiye; (ii) elucidate the phylogeographic relationships within *D. laniger* and *D. nitedula* using both mitogenomes and mitochondrial cytochrome *b* (*CYTB*) sequences; and (iii) reconstruct the phylogenetic relationships among extant members of the suborder Sciuromorpha. Sixteen new mitogenomes were sequenced from Turkish samples, containing 37 genes (2 ribosomal *RNA*s, 13 protein‐coding genes, 22 transfer *RNA*s), exhibiting similarity to those of other Gliridae and Sciuridae species. Based on mitogenomic data, Bayesian Inference and Maximum Likelihood phylogenetic analyses revealed two major phylogroups corresponding to the two families, Gliridae and Sciuridae, which were both monophyletic. Analyses of mitogenomic and *CYTB* sequences revealed at least two major lineages (i: Anatolia and ii: Lesser Caucasus and Alborz) of *D. nitedula* in the Anatolian region of Türkiye. The mitochondrial *CYTB* data indicated that *D. laniger* exhibited at least two major lineages (Eastern and Western), whereas *D. nitedula* comprised multiple lineages and sublineages. The mean genetic distance between the two mitogenomic lineages of *D. nitedula* was 7.69%. Based on the *CYTB* data, the mean genetic distance between the Eastern and Western lineages of *D. laniger* was 7%, whereas the mean genetic distances among the lineages of *D. nitedula* ranged from 6% to 13%. Major lineages of both *D. laniger* and *D. nitedula* might be considered distinct species throughout the species' range. This study demonstrates that complete mitogenomes for reconstructing the Gliridae phylogeny provides important information for revealing phylogenetic and phylogeographic relationships.

## Graphical abstract

This study presents mitogenomic analyses of three squirrel (Sciuridae) and three dormouse (Gliridae) species from Türkiye, uncovering significant phylogenetic and phylogeographic insights within the suborder Sciuromorpha. Phylogenetic analyses positioned Gliridae as the basal group within the suborder, and mitogenomic and *CYTB* data revealed distinct genetic lineages for *Dryomys laniger* and *Dryomys nitedula* , suggesting potential species‐level distinctions. These findings underscore the value of complete mitogenomes in understanding the evolutionary and taxonomic relationships in Gliridae.

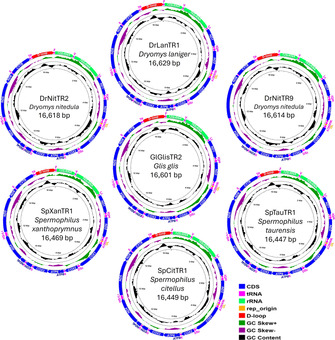

## 1 Introduction

Evolutionary studies based on molecular markers, such as mitochondrial and nuclear DNA, have revealed phylogeographic patterns that enable the inference of phylogenetic relationships and the estimation of evolutionary histories for various rodent groups (Montgelard et al. 2008; Huchon et al. 2002; Blanga‐Kanfi et al. 2009; Fabre et al. 2013; Steppan and Schenk 2017; D'Elía et al. 2019; Swanson et al. 2019; etc.). Rodentia, the most speciose order of living mammals, comprises five suborders: Sciuromorpha, Castorimorpha, Myomorpha, Anomaluromorpha, and Hystricomorpha (Carleton and Musser 2005). The modern fauna of the suborder Sciuromorpha includes at least 328 species within 72 genera, grouped into three families; Aplodontiidae, Gliridae, and Sciuridae (Carleton and Musser 2005; Burgin et al. 2018). Seven species of Gliridae and five species of Sciuridae, inhabiting various habitats, are distributed in Türkiye (Kryštufek and Vohralík 2005, 2009).

The utilization of genetic markers such as chromosomes, mitochondrial DNA, and nuclear DNA has led to revisions of the evolutionary relationships and taxonomic status of various glirid and sciurid taxa in Türkiye (Doğramacı et al. 1994; Gündüz et al. 2007; Grigoryeva et al. 2015; Kankılıç et al. 2018; Aghbolaghi et al. 2019, 2020; Demirtaş 2022; İbiş et al. 2022). Over the last two decades, one sciurid taxon ( *Spermophilus taurensis* , Taurus ground squirrel) was described by Gündüz et al. (2007) in the Anatolian part of Türkiye. Although sequences of individual mitochondrial genes for most Turkish sciuromorphs (Gündüz et al. 2007; Demirtaş 2022; İbiş et al. 2022) are available in the GenBank database, complete mitochondrial genome sequences of Turkish sciuromorphs are available only for *Sciurus anomalus* (Caucasian squirrel), *S. vulgaris* (Red squirrel) and *S. taurensis* within the family Sciuridae (İbiş et al. 2022; Matrosova et al. 2023).

The mitochondrial genome (mitogenome) is a molecular marker with features such as haploid (maternal) inheritance, lack of recombination, and high variation due to a relatively higher rate of DNA substitution compared to single‐copy nuclear DNA sequences (Brown et al. 1979). In the last two decades, complete mitochondrial sequences have been used in mitogenome characterization, molecular phylogenetics, and evolutionary studies, including those focused on sciuromorph taxa (Reyes et al. 2000; Ryu et al. 2013; Chao et al. 2014; Cong et al. 2017; De Abreu Jr et al. 2020; Boukhdoud et al. 2021; Forcina et al. 2022; İbiş et al. 2022; Matrosova et al. 2023; Zhao et al. 2024). The availability of complete mitogenomes as a molecular marker facilitates studies that aim to reconstruct the evolutionary relationships within the suborder Sciuromorpha. In this context, the addition of new mitogenomes for Sciuromorpha may contribute not only to reconstructing the phylogeny of the suborder but also to estimating divergence times. Although evolutionary studies have improved our understanding of intra‐ and inter‐specific relationships, the evolutionary relationships of certain populations or species within the genus *Dryomys* remain undetermined. Specific attention should be given to the populations of the forest dormouse, *Dryomys nitedula* , which is widely distributed in Eurasia. As many as 24 subspecies have been described for this taxon, some of which have been suggested to represent different species, but there remains no consensus on their taxonomic validity (Kryštufek and Vohralík 2005; Mohammadi et al. 2021). Furthermore, unresolved taxonomic issues within Sciuromorpha are not specific to the *Dryomys* populations. Other taxa within the families Gliridae and Sciuridae require further investigation due to their phylogeographic structure and potential cryptic diversity (Gündüz et al. 2007; Kryštufek et al. 2021; Mori et al. 2024). Addressing these taxonomic uncertainties is critical for understanding the biodiversity and evolutionary history of Sciuromorpha.

Here, we have sequenced and annotated 16 new complete mitogenomes of sciuromorph species from Türkiye. The current study aims to: (i) perform mitogenome characterization (gene arrangement, gene content, codon usage, and tRNA secondary structures) for three sciurid ( *Spermophilus citellus* , *S. taurensis* , and *S. xanthoprymnus* ) and three glirid ( *Glis glis* , *D. nitedula* , and *D. laniger* ) species from Türkiye, (ii) reveal phylogeographic relationships of populations within *D. laniger* and *D. nitedula* using evolutionary signals derived from both mitogenomes and mitochondrial cytochrome *b* (*CYTB*) sequences, and (iii) reconstruct phylogenetic relationships among the extant members of the suborder Sciuromorpha.

## 2 Materials and Methods

### 2.1 Ethics Statement, Sampling, and DNA Extraction

**FIGURE 1:**
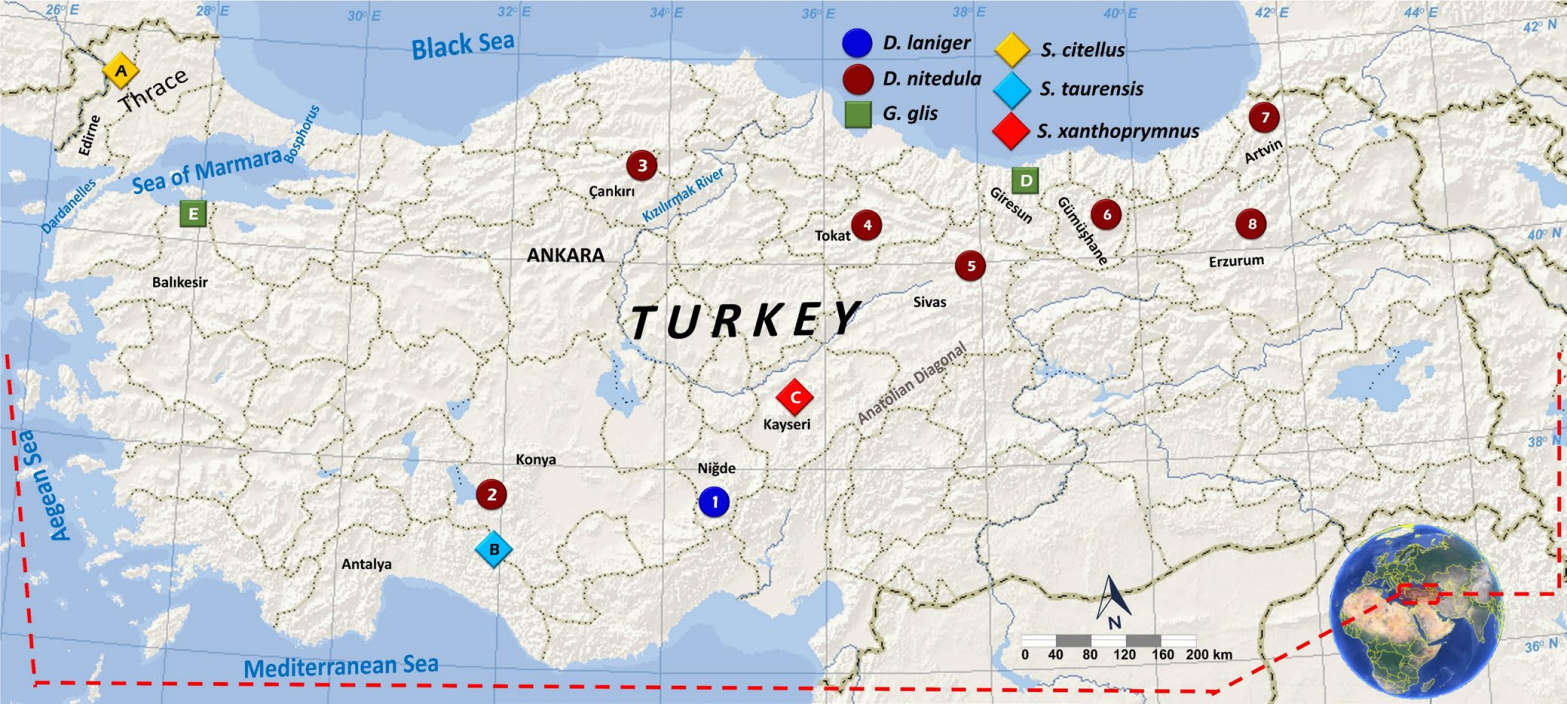
A map showing collection localities for Turkish samples belonging to Gliridae and Sciuridae.

The Local Ethical Committee of Laboratory Animal Experimentation at Erciyes University approved all experiments involving Turkish sciuromorphs (Protocol Nr.: 23/061, Date: April 05, 2023). Tissues (kidney and muscle) were retrieved from specimens belonging to species of Gliridae ( *G. glis* , *D. nitedula* , and *D. laniger* ) and Sciuridae ( *S. citellus* , *S. xanthoprymnus* , and *S. taurensis* ) preserved in the mammalian collection of Erciyes University in Kayseri, Türkiye (Figure 1, Table 1). Genomic DNA (gDNA) was extracted using the DNeasy Blood & Tissue Kit (QIAGEN, Germany) following the manufacturer's protocol. The gDNA of all samples was standardized to ~25 ng/μL with sterile dH_2_O and stored at −20°C until Long‐Range PCR amplification. Quality and concentration of the gDNA were assessed using the Qubit dsDNA BR Assay Kit with the Qubit 2.0 Fluorometer Quantitation Platform (Invitrogen, Thermo Fisher Scientific), and 1% agarose gel electrophoresis stained with ethidium bromide.

**TABLE 1:** A list of Turkish samples belonging to Gliridae and Sciuridae from which new complete mitogenomes were sequenced and assembled.

Map code in Figure 1	Collection/accession number	Taxon name	Haplotype code	Mitogenome length (bp)	Location
1	1899/PQ533836	*Dryomys laniger*	DrLanTR1	16.629	Ulukışla, Niğde, Türkiye
2	1153/PQ533837	*Dryomys nitedula*	DrNitTR1	16.618	Beyşehir, Konya, Türkiye
3	754/PQ533838	*D. nitedula*	DrNitTR2	16.618	Ilgaz, Çankırı, Türkiye
3	1887/PQ533843	*D. nitedula*	DrNitTR3	16.619	Ilgaz, Çankırı, Türkiye
4	1890/PQ533839	*D. nitedula*	DrNitTR4	16.620	Tokat, Türkiye
5	1155/PQ533841	*D. nitedula*	DrNitTR5	16.620	Zara, Sivas, Türkiye
5	1156/PQ533840	*D. nitedula*	DrNitTR6	16.619	Zara, Sivas, Türkiye
6	1154/PQ533842	*D. nitedula*	DrNitTR7	16.620	Arzular, Gümüşhane, Türkiye
7	1833/PQ533844	*D. nitedula*	DrNitTR8	16.617	Karagöl, Artvin, Türkiye
8	1878/PQ533845	*D. nitedula*	DrNitTR9	16.614	Dumlu, Erzurum, Türkiye
8	1879/PQ533846	*D. nitedula*	DrNitTR9	16.614	Dumlu, Erzurum, Türkiye
A	336/PQ533849	*Spermophilus citellus*	SpCitTR1	16.449	Edirne, Türkiye
B	339/PQ533850	*Spermophilus taurensis*	SpTauTR1	16.447	Seydişehir, Konya, Türkiye
C	250/PQ533851	*Spermophilus xanthoprymnus*	SpXanTR1	16.469	Kayseri, Türkiye
D	1831/PQ533847	*Glis glis*	GlGlisTR1	16.602	Giresun, Türkiye
E	1832/PQ533848	*G. glis*	GlGlisTR2	16.601	Kapıdağ, Balıkesir, Türkiye

### 2.2 Long‐Range PCR Amplification, Sequencing Library, Mitogenome Assembly, and Annotation

Complete mitogenomes of Turkish sciuromorphs were amplified by Long‐Range PCR using the NEB LongAmp Taq 2× Master Mix (M0287S; New England Biolabs, USA) following the manufacturer's protocol. Two primer pairs (CrocAL1‐2024L‐CrocBH1‐13002H and ScVu‐11712L‐LuLu‐2503H, İbiş et al. 2022) were used to generate Long‐Range PCR products for two overlapping mitogenome fragments (~11 and ~7.5 kb, respectively). The reaction mixture and thermal cycling profile for long‐range PCR amplifications were prepared according to the study of İbiş et al. (2022). A negative control (no gDNA) was included during all PCR procedures to detect possible contamination. A 1% agarose gel was used to run 5 μL of the PCR product to check for amplification success.

Quantification of the PCR products was performed using a Qubit dsDNA BR Assay Kit (Cat. No: Q32850; Invitrogen, Thermo Fisher Scientific, USA). The PCR products were diluted to a concentration of 0.2 ng/μL with sterile dH_2_O, and the sequencing library was prepared using 1 ng of amplicon (5 μL in volume). Purification of the PCR products was performed using an AMPure XP Kit (Beckman Coulter, USA).

The Nextera XT DNA Library Prep Kit (Illumina, San Diego, USA) and Nextera XT DNA Library Preparation Index Kit v2 Set A (Cat. No: FC‐131‐2001; Illumina, San Diego, USA), were used to construct DNA libraries for each sample with index sequences, following the manufacturer's protocols. The quantity of each DNA library was normalized with bead‐based normalization. Mitogenome sequencing of Turkish sciuromorph samples with concentrations and volumes qualified with the Qubit dsDNA HS Kit was performed using a MiSeq Reagent Kit v2 (500 cycles) (Illumina) and a MiSeq platform at the Genome and Stem Cell Center—GenKok (Erciyes University, Kayseri, Türkiye). Approximately 500,000 paired‐end clean reads of 250 bp were obtained for each sample. The raw sequence reads were extracted in the FASTQ format by verifying with Geneious Prime 2021.0.1 software (Kearse et al. 2012), which was also used to filter and process the reads. The BBDuk Trimming Tool in Geneious Prime was used to perform trimming of Illumina adapters and quality filtering operations related to short reads (< 30 bp) and low‐quality bases (*Q*‐score < 20) in the raw sequences. The mitogenomes of the Turkish sciuromorph samples were assembled by means of the Geneious Mapper Algorithm and using the mitogenome of *Sciurus vulgaris* (GenBank accession number: NC_002369; Reyes et al. 2000) as the reference, with the following parameters: sensitivity: highest sensitivity/medium, fine tuning: iterate up to 25 times. The reads were also de‐novo assembled with the GetOrganelle toolkit using default parameters (Jin et al. 2020). The filtered and trimmed sequence reads were then remapped to the de‐novo contigs using BBMap tool (using the Normal Sensitivity parameter) in Geneious Prime to ensure coverage and completeness. tRNA secondary structure, and the gene borders for each mitogenome were checked using the MITOS2 (Donath et al. 2019) web server and manually edited. The Codon Usage webserver (https://www.bioinformatics.org/sms2/codon_usage.html) and Geneious Prime were used to calculate the number and frequency of each codon type and amino acid groups for the 13 protein‐coding genes (PCGs) in the Turkish sciuromorph mitogenomes. The repeated motifs in the D‐loop region (control region) were determined using the Tandem Repeats Finder Web Server (Benson 1999). Two formulas, [A−T]/[A+T] and [G−C]/[G+C], were used to carry out AT and GC Skew analyses, respectively.

### 2.3 Phylogenetic Analyses

Detailed information about the sequences of the Sciuromorpha taxa obtained from the GenBank database (NCBI; www.ncbi.nlm.nih.gov/genbank/) is presented in File S1. Phylogenetic analyses were carried out using two datasets, consisting of 121 complete mitogenomes (22 from Gliridae and 99 from Sciuridae with 14,401 bp in length) and 134 mitochondrial *CYTB* sequences (1140 bp in length) from Gliridae (Table 1, File S1). The MAFFT algorithm (Katoh et al. 2002) in Geneious Prime was used to align sequences for the mitogenome and *CYTB* datasets with default parameters (Algorithm: Auto, Scoring matrix: 200 PAM/K = 2, Gap open penalty: 1.53, Offset value: 0.123). Ambiguously aligned sequences within the mitogenome dataset were trimmed using Gblocks v.0.91b (Castresana 2000), with default parameters. DnaSP Ver. 5.10.01 (Librado and Rozas 2009) was used to determine haplotype diversity, nucleotide diversity (*π*), polymorphic sites, number of parsimony informative sites, and mitochondrial haplotypes in the *CYTB* dataset, particularly for within the species *Dryomys laniger* and *D. nitedula* .

For the phylogenetic analyses, we employed Bayesian Inference (BI) and Maximum Likelihood (ML). For these analyses, GTR+G+I was selected as the best‐fitting nucleotide substitution model for the two datasets by using the jModeltest v.2.1.10 program (Darriba et al. 2012) based on two criteria: the corrected Akaike Information Criterion and the Bayesian Information Criterion. The BI analysis was performed using MrBayes v.3.2.6 (Ronquist et al. 2012). Four MCMC (Markov Chain Monte Carlo) chains were iterated for 6 and 3 million generations and sampling every 1000th generation for the mitogenomic and the *CYTB* datasets, respectively. After applying a burn‐in of 10% (after reaching a mean standard deviation of split frequencies < 0.1), a 50% majority rule consensus tree with ≥ 95% Bayesian credibility intervals that were estimated with posterior probabilities was generated by using the remaining samples of the posterior distribution. FigTree v1.3.1 (Rambaut 2009) and Geneious Prime were used to visualize a tree diagram generated by the Bayesian analysis. The ML analysis was carried out by using MEGA11 (Tamura et al. 2021), with 1000 and 10,000 bootstrap replicates for the mitogenome and the *CYTB* datasets, respectively. In all the analyses, sequences of three species from the order Lagomorpha ( *Lepus europaeus* : This study, *Ochotona princeps* : AJ537415/NC_005358, and *Oryctolagus cuniculus* : AJ001588/NC_001913) were used as outgroups for tree rooting purposes. The Kimura 2‐Parameter (K2P) (Kimura 1980) model, with a bootstrap method by means of MEGA11 (Tamura et al. 2021), was employed to calculate pair‐wise genetic distances among haplotypes of sciuromorph sequences.

## 3 Results

### 3.1 New Mitogenomes for Glirid and Sciurid Species from Türkiye

A total of 16 new mitochondrial genomes (mitogenomes) belonging to *D. laniger* (1 sample), *D. nitedula* (10 samples), *G. glis* (2 samples), *S. citellus* (1 samples), *S. taurensis* (1 samples), and *S. xanthoprymnus* (1 samples) within the families Gliridae and Sciuridae were successfully sequenced using the next‐generation sequencing platform with a minimum coverage of 2300× and a maximum coverage of 8800×. The mitogenomes ranged from 16,447 to 16,620 bp in length, and the differences were attributed to insertion–deletion and tandem repeat element variations in the D‐loop region (control region), rRNAs, and tRNAs (Table 1). The new mitogenomes have been deposited into Genbank (accession numbers: PQ533836–PQ533851).

### 3.2 Characterization of New Mitogenomes from Glirid and Sciurid Species

**FIGURE 2:**
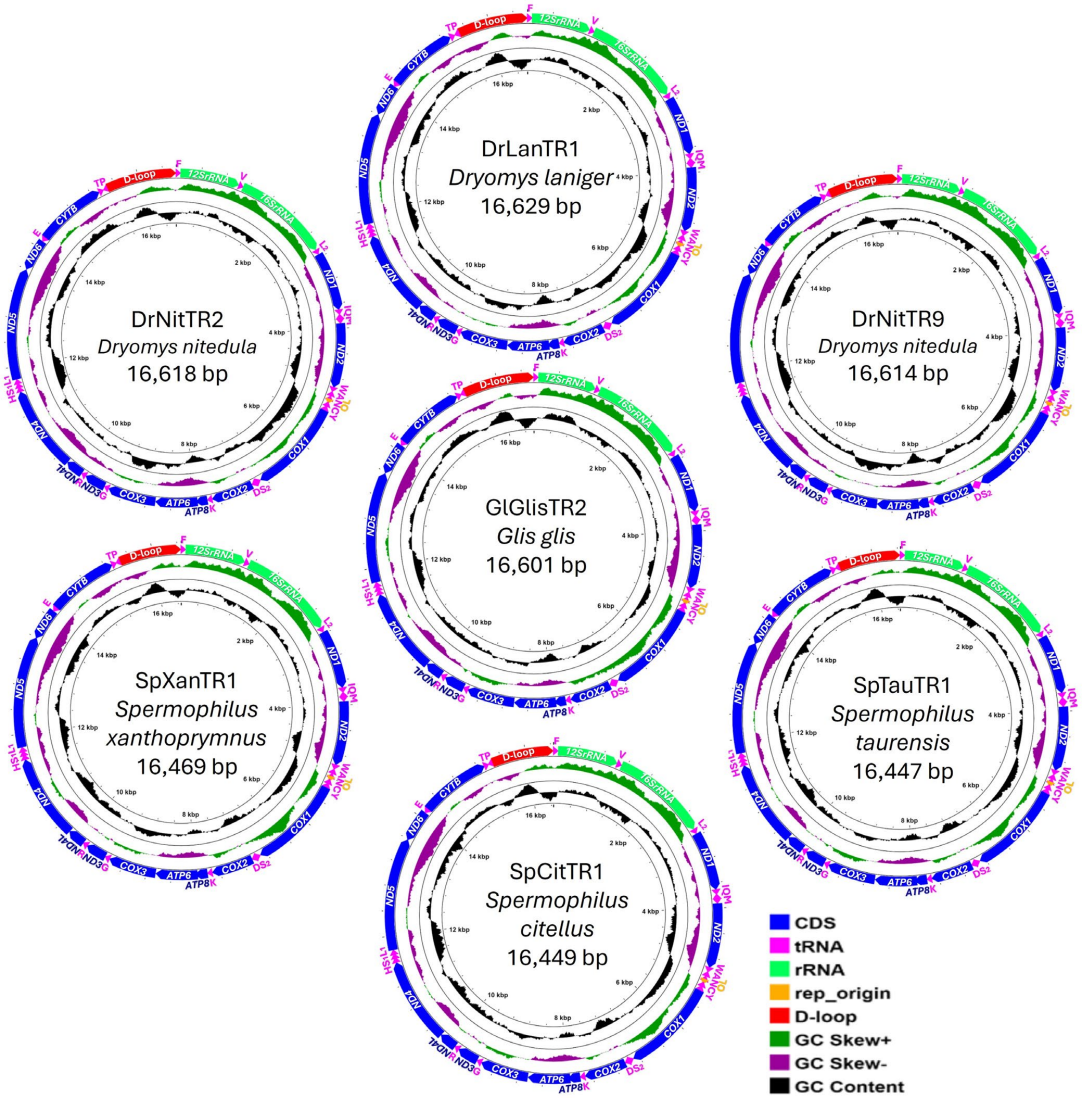
The circular graphical maps of the annotated mitogenomes for *Dryomys laniger* , *D. nitedula* (two samples), *Glis glis* , *Spermophilus citellus* , *S. taurensis* , and *S. xanthopyrmnus* from Türkiye.

Mitogenomes for each Turkish species within Gliridae and Sciuridae ( *D. nitedula* [DrNitTR1–DrNitTR9] *D. laniger* [DrLanTR1], *G. glis* [GlGlisTR1–GlGlisTR2], *S. citellus* [SpCitTR1], *S. taurensis* [SpTauTR1], and *S. xanthoprymnus* [SpXanTR1]) contained 13 Protein‐Coding Genes (PCGs), 22 transfer RNAs (tRNAs), two ribosomal RNAs (rRNAs), a non‐coding region (D‐loop/control region), and an origin of the light strand replication (*O*_L_). The gene order and organization were relatively similar to those of previously published Gliridae and Sciuridae mitogenomes. Of the 37 genes, 28 (14 tRNAs, 12 PCGs, and two rRNAs) were encoded on the Heavy strand and nine genes (8 tRNAs and 1 PCG) on the Light strand (Figure 2, File S2).

### 3.3 Nucleotide Composition

The nucleotide composition of mitogenomes for each species in Gliridae and Sciuridae are presented in File S3. Adenine (A) had the highest percentage in nucleotide composition, while Guanine (G) had the lowest (A>T>C>G). The A+T content ranged from 60.9% to 64.1% in the Gliridae and from 61.7% to 64.6% in the Sciuridae mitogenomes. The A+T content was higher compared to the G+C content in the PCGs, tRNAs, rRNAs, and D‐loop regions. Based on Skew analysis, A/T skews were observed to be positive across the entire mitogenomes, the tRNAs, and the rRNAs of all species examined. Additionally, positive A/T skews were identified in the protein‐coding genes (PCGs) of *D. laniger* and *D. nitedula* , as well as in the D‐loop regions of *D. laniger* , *D. nitedula* , and *G. glis* . However, contrasting results were also observed, as A/T skews were found to be negative in the PCGs of *G. glis* , as well as in those of the *Sciurus* and *Spermophilus* species. Furthermore, negative A/T skews were identified in the D‐loop regions of the *Sciurus* and *Spermophilus* species (File S3).

### 3.4 PCGs and Codon Usage

For 10 out of the 13 PCGs (*ATP6*, *ATP8*, *COX1*, *COX2*, *COX3*, *ND1*, *ND4L*, *ND4*, *ND6*, and *CYTB*), the start codon was identified as ATG. The start codons of the *ND2*, *ND3*, and *ND5* genes were ATC or ATT, ATT or ATA, and ATA or ATT, respectively. Across the majority of the PCGs in the six species, TAA emerged as the most prevalent stop codon. However, a limited number of PCGs utilized TAG and AGA, or the incomplete forms T‐ or TA‐, as alternative stop codons (File S2).

Comparison of codon usage in PCGs of the mitogenomes of the six species are presented in File S4. In terms of codon usage frequency, Leucine (611 for DrLanTR1, 610 for DrNitTR2, 609 for DrNitTR9, 622 for GlGlisTR2, 610 for SpCitTR1, 605 for SpTauTR1 and 609 for SpTauTR1) and Isoleucine (345 for DrLanTR1, 340 for DrNitTR2, 338 for DrNitTR9, 359 for GlGlisTR2, 347 for SpCitTR1, 343 for SpTauTR1, and 355 for SpTauTR1) exhibited the highest occurrence for all Turkish species/lineages. Conversely, Cysteine had the lowest frequency, with 24 to 29 occurrences for species (25 for DrLanTR1, 24 for DrNitTR2, DrNitTR9, and GlGlisTR2, 28 for SpCitTR1 and SpTauTR1, and 29 for SpTauTR1). Among all amino acids, those with acidic properties constitute 4.3%–4.5%, those with basic properties 6.6%–6.8%, charged amino acids 10.90%–11.20%, polar uncharged amino acids 28.60%–29.40%, and hydrophobic amino acids 62.20%–62.80% (File S4).

### 3.5 Transfer (tRNA) and Ribosomal (rRNA) Genes

In the mitogenomes of species belonging to Gliridae and Sciuridae, the lengths of tRNAs ranged from 61 to 75 base pairs for glirid species and from 59 to 76 base pairs for sciurid species (File S2). No significant differences were observed in the secondary structure predictions of tRNAs among the six species' mitogenomes. All tRNAs displayed a typical clover‐leaf secondary structure, with the exception of tRNASer (AGY), which lacked a dihydrouridine (DHU) loop and stem in the tRNA Serin1 (S1)‐GCT structure (File S5). The *16S* and *12S* rRNAs were encoded on the Heavy strand and showed variations in length among the species (File S2).

### 3.6 D‐Loop and *O*_L_ ‐Replication Region

The D‐loop (control region) of the mitogenomes in Gliridae and Sciuridae, located between tRNA^*Pro*^ and tRNA^*Phe*^, had a length ranging from 1136 to 1158 bp and from 1000 to 1009 bp, respectively. No tandem repeat elements were detected in Turkish glirid and sciurid species, except for two species ( *D. nitedula* and *S. taurensis* ). In *D. nitedula* sequences, a tandem repeat element of 26 bp was detected 2.2 times, while in *S. taurensis* a tandem repeat element of 21 bp was observed two times. The Origin of Light‐strand Replication (*O*_L_) within the WANCY region constituted a noncoding mtDNA segment with a length of 30 bp ( *S. citellus* , *S. taurensis* , *S. xanthopyrmnus*, and *G. glis* ); 31 bp ( *D. nitedula* ); or 32 bp ( *D. laniger* ) (Figure 2, File S2). Additionally, the stem‐loop structure of the *O*_L_ in six species started with 5′‐TCTCC‐3′.

### 3.7 Intergenic Spacers and Overlap Regions

Both Gliridae and Sciuridae mitogenomes contained intergenic spacers and overlapping regions. The longest intergenic region (31 bp for Gliridae and 43 bp for Sciuridae) is between *ATP8* and *ATP6*, and there is a conserved region between the two genes. In the mitogenomes of the Sciuridae species, there are 8 overlapping regions and 13 intergenic spacers. However, there are eight overlapping regions and 14 or 15 intergenic spacers within the Gliridae species (File S2).

### 3.8 Phylogenetic Analyses

#### 3.8.1 Mitogenome Phylogeny of Sciuromorpha

**FIGURE 3:**
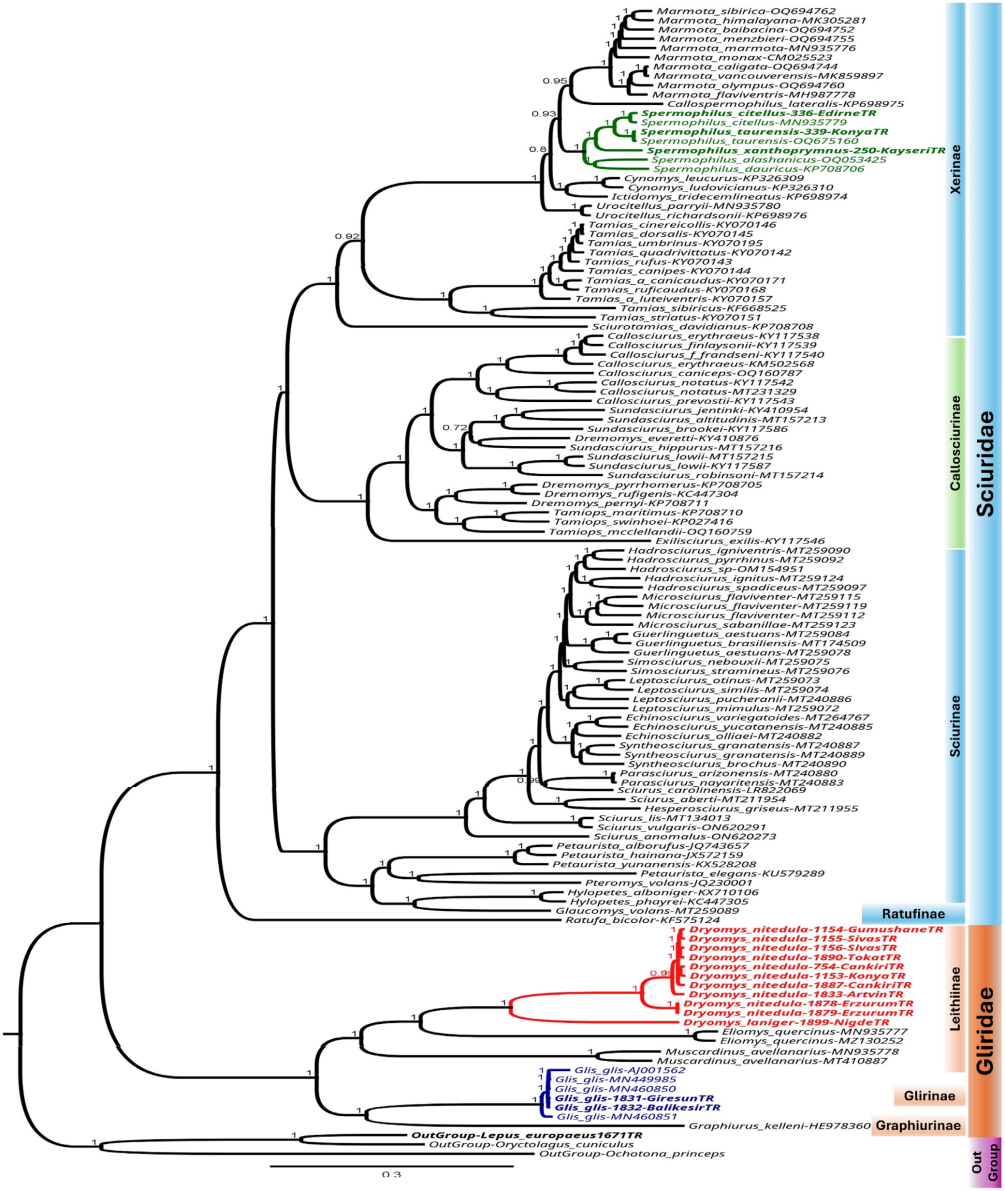
Bayesian phylogenetic tree reconstructed using mitogenomes for Sciuromorpha (Gliridae and Sciuridae) based on the GTR+G+I model and 6 million MCMC generations after removal of 10% burn‐in.

Based on complete mitogenomes (14,401 bp in length) of species belonging to Gliridae and Sciuridae and three outgroup species (Table 1, File S1), phylogenetic analyses performed using the GTR+I+G model of nucleotide substitution and BI and ML methods yielded similar topologies with high nodal support values. The phylogenetic trees consisted of two major phylogroups corresponding to the two families, Gliridae and Sciuridae, which were both monophyletic. The branch leading to the family Gliridae was separated as the first phylogroup within Sciuromorpha after the outgroup branch (Figure 3, File S6). The phylogenetic relationships among the Sciuridae showed that the genus *Spermophilus* was monophyletic and sister to a clade containing *Urocitellus* + (*Cynomys* + *Ictidomys*). *Spermophilus dauricus* was a sister species to *S. alashanicus* , whereas *S. citellus* was a sister species to *S. taurensis* . The Turkish *S. citellus* was clustered with a specimen from Central Europe (MN935779). *S. xanthoprymnus* was connected as a sister branch to *S. citellus* + *S. taurensis* (Figure 3, File S6). The phylogenetic relationships of the other members of Sciuridae that include the *Sciurus* species in Türkiye were emphasized in our previous study (İbiş et al. 2022), the results of which are consistent with the results of the current study.

The phylogenetic relationships within Gliridae showed that the subfamilies Glirinae and Leithiinae were each monophyletic. The subfamily Glirinae included two species belonging to two genera, *Glis* and *Graphiurus* ( *G. glis* + *Graphiurus kelleni* ). The samples of the Turkish *G. glis* were grouped together and placed into the same haplogroup with the *G. glis* samples from Southwestern Germany (MN449985), Wien‐Austria (MN460850), Montpellier‐France (AJ001562), and southern Italy (MN460851).

The other subfamily Leithiinae comprised four species belonging to three genera; *Dryomys*, *Eliomys* and *Muscardinus*; [(( *D. laniger* + *D. nitedula* ) + *Eliomys quernicus*) + *Muscardinus avellanarius* ]. The monophyly of the genus *Dryomys* was well supported, within which the *D. nitedula* mitogenomes were clustered together with high nodal support values, and *D. laniger* was sister to *D. nitedula* . The Turkish *D. nitedula* mitogenomes were divided into two major lineages: the Anatolia lineage contained samples from the Central Anatolian region (Çankırı, Konya, and Sivas) and the Central and East Black Sea regions (Gümüşhane and Tokat, and Artvin), whereas the Lesser Caucasus and Alborz lineage included samples from Erzurum in the Eastern Anatolian region (Figure 3, File S6).

Based on the K2P pairwise distances obtained from the mitogenomic dataset, the dissimilarity between the Turkish *S. citellus* (SpCitTR1/Accession number: PQ533849) and the Austrian *S. citellus* (MN935779) was 1.4%, whereas the dissimilarity between the two mitogenomes of the Turkish *S. taurensis* (SpTauTR1/Accession numbers: PQ533850 and OQ675160) was 0.1%. Moreover, the dissimilarity between *S. citellus* and *S. taurensis* was 4.9%, whereas *S. xanthopyrmnus* (SpXanTR1/Accession number: PQ533851) differed by ~8.4% from both *S. citellus* and *S. taurensis* . Sequence distances between *D. nitedula* and *D. laniger* ranged from 18.72% to 19.03%, with an average of 18.84%. Genetic distances among the *D. nitedula* mitogenomes ranged from 0.00% to 7.80%. Two mitogenomes of *D. nitedula* from Erzurum shared the same haplotype (DrNitTR9). Mean genetic distance between the two lineages of *D. nitedula* , Anatolia and Lesser Caucasus and Alborz, was 7.69%. The sequence dissimilarity between the two mitogenomes of *G. glis* from Balıkesir and Giresun was at least 0.02%. The dissimilarity between *G. glis* and *D. laniger* was 23.56%, whereas pairwise distances between *G. glis* and *D. nitedula* ranged from 23.80% to 24.27%, with an average of 24.11% (Table 2).

**TABLE 2:** Mean genetic distances among *Dryomys laniger* , *D. nitedula* , *Glis glis* , *Spermophilus citellus* , *S. taurensis* , and *S. xanthopyrmnus* from Türkiye based on mitogenomic data (A). Mean genetic distances among lineages proposed for *D. laniger* (B) and *D. nitedula* (C) based on mitochondrial *CYTB* data. *****: The same species and/or lineage; no value to compare.

A. Mean genetic distances based on mitogenomic data of six species			
	*D. laniger*	*D. nitedula*	*G. glis*
***Dryomys laniger***	*****	0.1884 (18.84%)	0.2356 (23.56%)
***Dryomys nitedula***		*****	0.2411 (24.11%)
***Glis glis***			*****

	*S. citellus*	*S. taurensis*	*S. xanthopyrmnus*
***Spermophilus citellus***	*****	0.048 (4.8%)	0.084 (8.4%)
***Spermophilus taurensis***		*****	0.084 (8.4%)
***Spermophilus xanthopyrmnus***			*****

Sublineages of *Dryomys nitedula*	nitedula_1	nitedula_2
**nitedula_1**	*****	0.0769 (7.69%)
**nitedula_2**		*****

B. Mean genetic distances based on mitochondrial *CYTB* data of *Dryomys laniger*		
Lineages of *Dryomys laniger*	Eastern lineage	Western lineage
Eastern lineage	*****	0.070 (7%)
Western lineage		*****

Sublineages of Western lineage within *Dryomys laniger*	Western Laniger_1	Western Laniger_2
Western Laniger_1	*****	0.020 (2%)
Western Laniger_2		*****

C. Mean genetic distances based on mitochondrial *CYTB* data of *Dryomys nitedula*								
Lineages/sublineages of *Dryomys nitedula*	Lesser Caucasus and Alborz (B1)	C and E Great Caucasus (B2)	W Great Caucasus (B3)	Kopet Dagh (A1)	Zagroz (A2)	Anatolia	Europe (C1)	Central Iran (D)
**Lesser Caucasus and Alborz (B1)**	*****	0.069 (6.9%)	0.08 (8%)	0.081 (8.1%)	0.106 (10.6%)	0.093 (9.3%)	0.104 (10.4%)	0.128 (12.8%)
**C and E Great Caucasus (B2)**		*****	0.074 (7.4%)	0.084 (8.4%)	0.1 (10%)	0.103 (10.3%)	0.116 (11.6%)	0.136 (13.6%)
**W Great Caucasus (B3)**			*****	0.069 (6.9%)	0.089 (8.9%)	0.077 (7.7%)	0.098 (9.8%)	0.117 (11.7%)
**Kopet Dagh (A1)**				*****	0.06 (6%)	0.076 (7.6%)	0.099 (9.9%)	0.121 (12.1%)
**Zagroz (A2)**					*****	0.094 (9.4%)	0.111 (11.1%)	0.134 (13.4%)
**Anatolia**						*****	0.096 (9.6%)	0.115 (11.5%)
**Europe (C1)**							*****	0.131 (13.1%)
**Central Iran (D)**								*****

#### 3.8.2 Phylogenetic Analysis of Gliridae Based on *CYTB* Sequences

**FIGURE 4:**
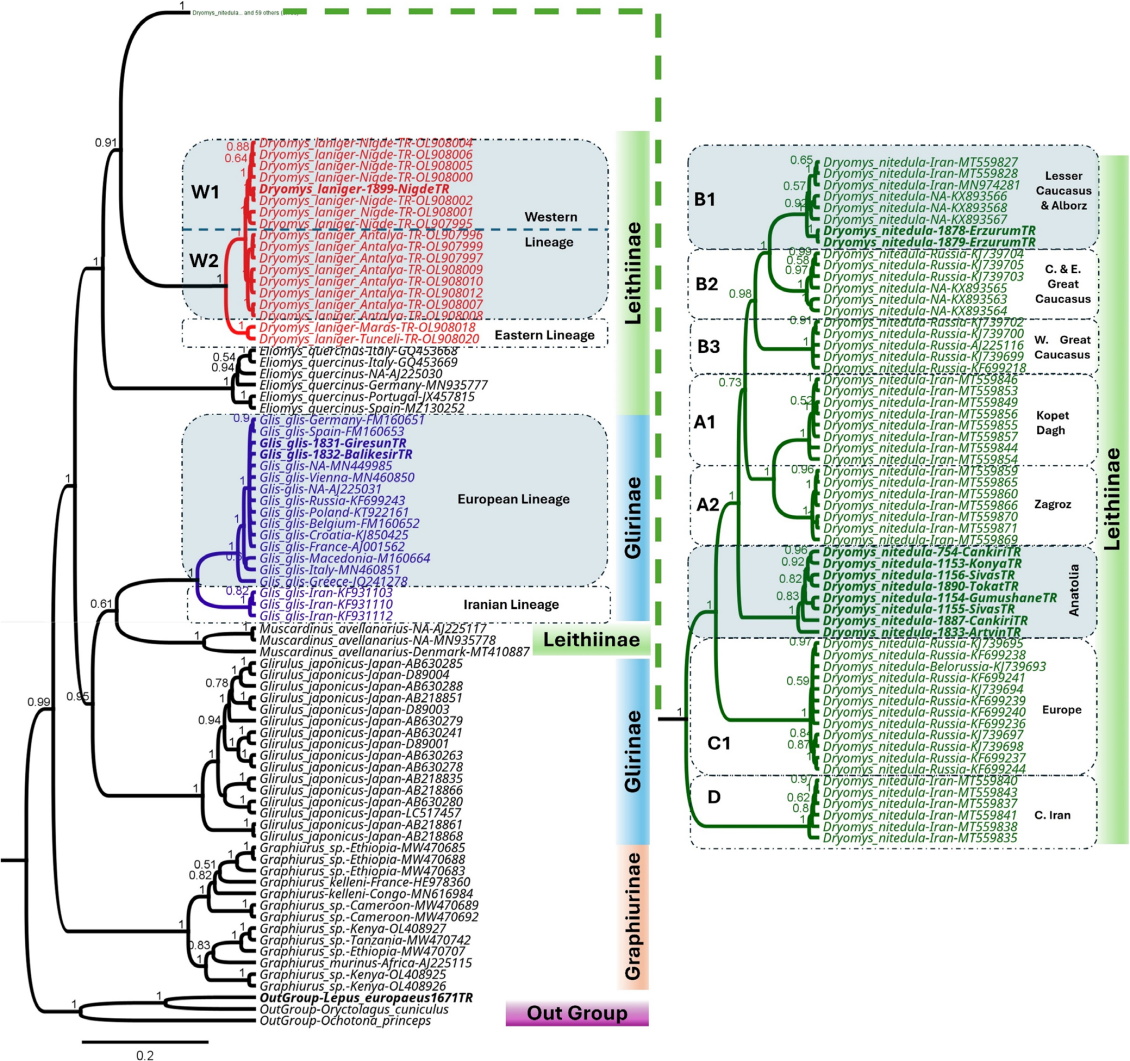
Bayesian phylogenetic tree reconstructed using mitochondrial *CYTB* sequences for Gliridae based on the GTR+G+I model and 3 million MCMC generations after removal of 10% burn‐in.

A total of 116 sequences were obtained from the NCBI database and this study for *D. laniger* (30) and *D. nitedula* (86) species for genetic analyses. Of these sequences, 18 different haplotypes were identified for *D. laniger* , while 59 different haplotypes were detected for *D. nitedula* . All the *CYTB* haplotypes of Turkish *Dryomys*, which were obtained this study, were not shared with sequences reported in previous studies. Haplotype diversities for *D. laniger* and *D. nitedula* were high (*h* = 0.936, SD = 0.029 and *h* = 0.985, SD = 0.005, respectively), and nucleotide diversities (*π*) were 0.03875 and 0.08067, respectively. A total of 130 and 309 polymorphic sites, 105 and 280 of which were parsimony informative, were identified in the *D. laniger* and *D. nitedula* sequences respectively (Figure 4, File S7).

BI and ML analyses of the *CYTB* dataset (1140 bp in length) for Gliridae using the GTR+I+G substitution model revealed relatively similar topologies (Figure 4, File S8). As was found in the mitogenome phylogeny (Figure 3, File S6), there was a close relationship between *Dryomys* and *Eliomys* when compared to other genera within Gliridae. A fundamental difference was observed in the positioning of species in the BI and ML trees. In the BI tree, *D. nitedula* and *D. laniger* were clustered together, with *Eliomys quercinus* branching as the basal node to these species. In contrast, in the ML tree, *E. quercinus* was grouped between *D. nitedula* and *D. laniger* , with *D. laniger* in the basal position relative to the other two species (Figure 4, File S8).

Haplotypes obtained from the distribution area of *D. nitedula* were divided into eight main lineages: (i) Kopet Dagh, (ii) Zagros, (iii) Lesser Caucasus and Alborz, (iv) Central and Eastern Great Caucasus, (v) Western Great Caucasus, (vi) Eastern Europe, (vii) Central Iran, and (viii) Anatolia. The Turkish haplotypes of *D. nitedula* were grouped into two main lineages: (iii) Lesser Caucasus and Alborz included samples from Georgia, Iran and Erzurum, Türkiye, and (viii) Anatolia only comprised samples from the central Anatolian and the eastern Black Sea regions of Türkiye (Figure 4, File S8). This split between the Turkish samples of *D. nitedula* was also observed in the mitogenome phylogeny.

Haplotypes of *D. laniger* that are endemic to the Anatolian region of Türkiye were clustered into two main lineages: The Eastern lineage included samples from Kahramanmaraş and Tunceli, and the Western lineage contained samples from Niğde and Antalya. Moreover, two sublineages comprised the Western lineage: Western Laniger_1 (W1), including samples from Antalya, and Western Laniger_2 (W2), containing samples from Niğde (Figure 4, File S8). The *D. laniger* lineages might have evolved due to various barriers such as the Göksu River and Seyhan River, and such phylogeographic breaks may be a result of long‐standing barriers to gene flow within the distribution area of *D. laniger* . The Seyhan River might possibly be a barrier between the Western lineage (containing samples from Niğde and Antalya on the western side of the river) and the Eastern lineage (including samples from Kahramanmaraş and Tunceli on the eastern side of the river). In addition, the Göksu River might possibly be a barrier between the two Western sublineages of *D. laniger* ; Western Laniger_1 (W1) including samples from Antalya on the western side of the river and Western Laniger_2 (W2) containing samples from Niğde on the eastern side of the river.

Mean genetic distances among the *D. nitedula* lineages ranged from 6% to 13.6%. The lowest mean distance (6%) was between the Kopet Dagh (A1) lineage and the Zagros (A2) lineage, whereas the highest mean distance (13.6%) was between the C and E Great Caucasus (B2) lineages and the Central Iran (D) lineage. The mean genetic distance between the Western lineage and the Eastern lineage of *D. laniger* was 7%, ranging from 6.5% to 8.2%. The mean genetic distance between of the two Western sublineages of *D. laniger* (Western Laniger_1 and Western Laniger_2) was 2% (Table 2).

The haplotypes of *G. glis* were clustered into two main lineages: European and Iranian. The European lineage comprised the haplotypes from Austria, France, Germany, Spain, Türkiye, Russia, Poland, Belgium, Croatia, Italy, Macedonia, Greece, and an unknown location, whereas the Iranian lineage included the haplotypes from Iran. We observed a polytomy for the haplotypes in the European lineage (Figure 4, File S8).

## 4 Discussion

The current study focused on characterizing the mitogenomes of the three Gliridae ( *D. laniger* , *D. nitedula* , and *G. glis* ) and the three Sciuridae ( *S. citellus* , *S. taurensis* and *S. xanthoprymnus* ) species from Türkiye, and estimating the phylogenetic relationships among the taxa within the suborder Sciuromorpha based on mitogenomes and also the taxa within the family Gliridae based on mitochondrial *CYTB* sequences. The findings from the current study reported the mitochondrial genomes of the species of Gliridae and Sciuridae found in Türkiye with regards to mitogenome structure, gene content, and codon usage. Furthermore, the estimated phylogenetic trees generated using BI and ML methods were generally consistent with those reported in previous studies and provided insights into the evolution of these families and the placement of the species that occur in Türkiye.

### 4.1 A Comparison of Turkish *Dryomys*, *Glis*, and *Spermophilus* Mitogenomes with Other Glirid and Sciurid Mitogenomes

Numerous studies have reported available mitogenomes for the genera within the families Gliridae and Sciuridae (Reyes et al. 2000; Fabre et al. 2013; Ryu et al. 2013; Chao et al. 2014; Cong et al. 2017; De Abreu Jr et al. 2020; Boukhdoud et al. 2021; Emser et al. 2021; Margaryan et al. 2021; Forcina et al. 2022; Matrosova et al. 2023; Zhao et al. 2024), and these studies demonstrated the usefulness of mitogenome analysis in characterizing the genetic diversity and evolutionary relationships within the two sciuromoph families.

Five glirid (*Dryomys*, *Eliomys*, *Glis*, *Muscardinus*, and *Myomimus*) and two sciurid genera (*Spermophilus* and *Sciurus*) are distributed in Türkiye (Kryštufek and Vohralík 2005, 2009). Although the mitogenomes for two species of the Turkish *Sciurus* ( *S. anomalus* and *S. vulgaris* ) (İbiş et al. 2022) and one species of the Turkish *Spermophilus* ( *S. taurensis* ) (Matrosova et al. 2023) have been reported previously, mitogenomes for *S. citellus* and *S. xanthoprymnus* from Türkiye were unavailable until now. However, the mitogenome of *S. citellus* (European ground squirrel) from Austria is available from the GenBank database (Emser et al. 2021). In the present study, the complete mitogenomes of the Turkish *S. citellus* , *S. taurensis* and *S. xanthoprymnus* were found to consist of different sequence lengths (16,449, 16,447, and 16,469 bp, respectively). This difference was also observed among the mitogenomes of *S. alashanicus* (Zhao et al. 2024), *S. citellus* (Emser et al. 2021), *S. dauricus* (Unpublished) and *S. taurensis* (Matrosova et al. 2023). The GenBank database contains mitogenomes of three glirid species: *Eliomys quercinus* from Germany and Spain; *G. glis* from Austria, France, and Italy; and *M. avellanarius* from Belgium and Denmark (see https://www.ncbi.nlm.nih.gov/genbank/; Reyes et al. 1998; Margaryan et al. 2021; Emser et al. 2021; Forcina et al. 2022). To these we have sequenced and added mitogenomes of the Turkish *G. glis* , *D. laniger* , and *D. nitedula* . As found in the mitogenomes of the Turkish *Spermophilus* species, the mitogenomes of the Turkish *G. glis* , *D. laniger* , and *D. nitedula* were also found to be of different sequence lengths (16,601 and 16,602 bp, 16,612 bp, and from 16,614 to 16,620 bp, respectively). The length variations observed among the species within the families Gliridae and Sciuridae were mainly due to insertion–deletion variations and the number of tandem repeats in the control region/D‐loop and in tRNAs (Reyes et al. 1998; Emser et al. 2021; Margaryan et al. 2021; Forcina et al. 2022; İbiş et al. 2022; Matrosova et al. 2023).

In the mitogenomes of the Turkish glirid and sciurid species, the most common nucleotides in the first and second positions of the PCG start codons were A and T, respectively. However, the most common nucleotides in the first and second positions of the PCG stop codons was T and A, respectively. The higher A+T versus G+C content is due to these nucleotide positions, and this was also observed in the mitogenomes of other species within Gliridae and Sciuridae (Reyes et al. 2000; Fabre et al. 2013; Ryu et al. 2013; Chao et al. 2014; Cong et al. 2017; De Abreu Jr et al. 2020; Boukhdoud et al. 2021; Emser et al. 2021; Margaryan et al. 2021; Forcina et al. 2022; Matrosova et al. 2023; Zhao et al. 2024). One possible explanation for the bias in base composition is the occurrence of asymmetric mutations during replication and transcription, as well as the subsequent selective pressures acting on these mutations (Cheng et al. 1992; Wei et al. 2010; Chen et al. 2020).

### 4.2 Mitogenome Phylogeny of Gliridae and Sciuridae within the Suborder Sciuromorpha

Phylogenetic analysis of mitogenomic sequences can provide valuable insights into the evolutionary relationships among different species and groups of organisms. The suborder Sciuromorpha includes the families Aplodontiidae, Gliridae, and Sciuridae (Wilson and Reeder 2005). The current study reconstructed the Sciuromopha phylogeny by combining our data with other mitogenomes of Gliridae and Sciuridae. However, due to the absence of mitogenomes, Aplodontiidae, as represented by the sole extant species *Aplodontia rufa* , endemic to western North America, was not included in the phylogenetic analyses. The mitogenome phylogeny of Sciuromopha resulted in two well‐supported main phylogroups, Gliridae and Sciuridae.

Numerous studies have used mitogenome sequences to reveal intra‐ and inter‐specific relationships within the families Gliridae and Sciuridae (Reyes et al. 2000; Fabre et al. 2013; Ryu et al. 2013; Chao et al. 2014; Cong et al. 2017; De Abreu Jr et al. 2020; Boukhdoud et al. 2021; Emser et al. 2021; Margaryan et al. 2021; Forcina et al. 2022; Matrosova et al. 2023; Zhao et al. 2024). However, studies revealing intra‐ and inter‐specific relationships based on mitogenomic data are still scarce for some species of Gliridae ( *D. laniger* and *D. nitedula* ) and Sciuridae ( *S. citellus* , *S. taurensis* , and *S. xanthoprymnus* ).

By using 23 mammalian mitogenomes, which included the complete mitogenome of the fat dormouse *G. glis* (Gliridae), Reyes et al. (1998) investigated the phylogenetic relationships among four rodent species (fat dormouse, guinea pig, rat and mouse) and found that *G. glis* was closer to the guinea pig (Caviidae, Caviomorpha) than to the rat and mouse (Muridae, Myomorpha). In another study by Reyes et al. (2000), *G. glis* was most closely related to *S. vulgaris* . The guinea pig was clustered with *G. glis* + *S. vulgaris* , and then the rabbit (order Lagomorpha) was clustered with ( *G. glis* + *S. vulgaris* ) + the guinea pig. The studies of Horner et al. (2007) and Horn et al. (2011) suggested *G. glis* + *S. vulgaris* as sister taxa, whereas the study by Fabre et al. (2013) suggested the relationship of *S. vulgaris* + ( *G. glis* + *Graphiurus kelleni* ). In two studies that included phylogenetic analyses within Rodentia and a revision of rodent phylogeny, *G. glis* was positioned in the basal position of the Sciuromorpha clade, that is, *G. glis* + [( *Pteromys volans* + *S. vulgaris* ) + *Marmota himalayana* ] (Ryu et al. 2013) and *G. glis* + ( *S. vulgaris* + *M. himalayana* ) (Chao et al. 2014). By using mitogenomes of seven species in Sciuridae and two species in Gliridae ( *G. glis* and *G. kelleni* ), Cong et al. (2017) constructed a ML phylogenetic tree where *G. glis* and *G. kelleni* were clustered together. Gliridae and Sciuridae were grouped as sister families in the study of Emser et al. (2021), where *G. glis* was recovered to be the sister lineage to the phylogroup including *Eliomys quercinus* + *M. avellanarius* , whereas *S. citellus* was recovered to be the sister lineage to the phylogroup group containing *Ictidomys tridecemlineatus* + ( *Urocitellus richardsonii* + *U. parryii* ) in Sciuridae. In the phylogenetic reconstruction of *E. quercinus* (European garden dormice) by Forcina et al. (2022) based on complete mitogenomes, *E. quercinus* and *M. avellanarius* were sister taxa, and *G. kelleni* was positioned as sister to *E. quercinus* + *M. avellanarius* , whereas *G. glis* was positioned as the most basal taxon. In two recent studies (Matrosova et al. 2023; Zhao et al. 2024), *S. alashanicus* and *S. dauricus* and *S. citellus* and *S. taurensis* were each found to be sister taxa, respectively.

BI and ML analyses in the current study resulted in phylogenetic trees with similar topologies. However, there were several relatively minor differences between the trees in terms of the positioning of branches corresponding to taxa or haplotypes within taxa. The phylogenetic status of most taxa within Sciuridae was discussed in detail in previous studies (De Abreu Jr et al. 2020; İbiş et al. 2022). In this context, here we did not re‐evaluate the phylogenetic status of other taxa belonging to Sciuridae, except for species within the genus *Spermophilus*, specifically *S. citellus* , *S. dauricus* , *S. taurensis* , and *S. xanthoprymnus* . *Spermophilus* was found to be a monophyletic genus within Sciuridae. The Turkish *S. citellus* was grouped with the Austrian *S. citellus* (Emser et al. 2021). *S. citellus* and *S. taurensis* were sister taxa as reported by Matrosova et al. (2023) and Zhao et al. (2024), and *S. xanthoprymnus* was recovered to be a sister lineage to *S. citellus* + *S. taurensis* . These results were consistent with the study of Gündüz et al. (2007), who reported the molecular phylogenetics of the genus *Spermophilus* in Türkiye using DNA sequences from the mitochondrial *CYTB*, D‐loop, and *tRN*As, and the *X* and *Y* chromosomes. The K2P sequence distance values among the Turkish *Spermophilus* species obtained from the mitogenomic data were also quite similar to the values reported by Gündüz et al. (2007) based on partial mitochondrial sequences.

In the BI and ML trees, genera within Gliridae (*Dryomys*, *Eliomys*, *Muscardinus*, *Glis*, and *Graphiurus*) were monophyletic. The tree branches corresponding to *G. glis* and *G. kelleni* formed the first dichotomy within the family. The Turkish samples of *G. glis* were grouped together with the *G. glis* samples from Southwestern Germany (MN449985, unpublished) and Wien–Austria (MN460850; unpublished). In the current study, the phylogenetic position of *G. glis* was compatible with the results of Fabre et al. (2013), Ryu et al. (2013), Chao et al. (2014), Cong et al. (2017), and Forcina et al. (2022). Moreover, the results of several studies also showed that *G. glis* and *G. kelleni* were sister or closely positioned taxa (Fabre et al. 2013; Cong et al. 2017; Forcina et al. 2022). The *Dryomys* species, *D. laniger* and *D. nitedula* , were sister taxa. In all the mitogenome trees (Figure 3, File S6), the branches corresponding to *D. laniger* + *D. nitedula* and *E. quercinus* (Emser et al. 2021; Forcina et al. 2022) formed a dichotomy. The *D. nitedula* haplotypes were grouped into two major lineages: the Anatolia lineage contained samples from the Central Anatolian region (Çankırı, Konya, and Sivas) and the Central and East Black Sea regions (Gümüşhane, Tokat, and Artvin), and the Lesser Caucasus and Alborz lineage included samples from Erzurum in the Eastern Anatolian region. The Lesser Caucasus and Alborz lineage, which included two samples from Erzurum that shared the same haplotype, was quite different from the Anatolia lineage, and the K2P sequence distance between these lineages was 7.69%. The Lesser Caucasus and Alborz lineage is genetically and geographically distinct from the other samples of *D. nitedula* , and it may be minimally considered an evolutionarily significant unit (Hoelzel 2023). Based on K2P distances for the *CYTB* gene, the limit of intra‐specific variation was suggested as ≥ 2.4% by Bradley and Baker (2001). However, there is no information about the lower and upper limits of inter‐specific mitogenome distances in mammals. The distance of 7.69% that was found between the Erzurum samples (Lesser Caucasus and Alborz lineage) and the other samples (Anatolia lineage) of *D. nitedula* from Türkiye can be considered a remarkable threshold value for possibly describing a new taxon within *D. nitedula* , but this will need to be further tested with sequences obtained from the nuclear genome. Nonetheless, based on the BI, ML, and K2P distance analyses, we tentatively propose that the Lesser Caucasus and Alborz lineage, which included the two Erzurum samples, may belong to a distinct species within the genus *Dryomys*.

### 4.3 Phylogeny of Gliridae Based on Mitochondrial *CYTB* Sequences

In the last decade, numerous studies have used mitochondrial and/or nuclear sequences to investigate intra‐ and inter‐specific relationships of taxa within the Gliridae, particularly species of the genus *Dryomys* ( *D. laniger* and *D. nitedula* ) from Türkiye and adjacent regions (Grigoryeva et al. 2015; Bisconti et al. 2018; Çetintaş et al. 2018, 2022; Kankılıç et al. 2018, 2019; Mohammadi et al. 2021; El Mojahid et al. 2022). Kryštufek and Vohralik (2009) reported that seven species and five genera of Gliridae are distributed in Türkiye. In a mitochondrial phylogeographic study based on *CYTB* sequences of samples obtained from the Caucasian and the Russian Plain regions (Grigoryeva et al. 2015), *D. nitedula* samples were clustered into two main lineages, originating from the Caucasus region and Eastern Europe, respectively. The genetic distance between the two lineages was found to be 9.94%. Moreover, the samples from the Caucasian region were also divided into two sublineages, representing the Central Caucasus versus Western Caucasus, and genetic distance between these two sublineages was 6%. Grigoryeva et al. (2015) emphasized that the genetic distance between the Caucasus and Eastern Europe (Russian Plain) lineages were a value corresponding to the genetic distance typically observed between mammalian species (Bradley and Baker 2001), and that the Western and the Central Caucasian sublineages can be considered sibling species due to the significant genetic distance value between them.

Genetic variation, molecular phylogeny and phylogeography of the Turkish *Dryomys* were investigated using mitochondrial *ND1* (Kankılıç et al. 2018) and 12S rRNA (Kankılıç et al. 2019) sequences, as well as sequences from intron 7 of the nuclear beta–fibrinogen gene (Kankılıç et al. 2019). In these studies, which used Bayesian, ML, and network analyses, *D. nitedula* samples were clustered into four main lineages; one in the Thracian region and three in the Anatolian region. According to the results of Kankılıç et al. (2018, 2019), the inferred Bayesian and ML topologies were partly different. Bayesian analysis revealed that *D. laniger* and *D. nitedula* were grouped together, whereas the ML analysis showed that *E. quercinus* was grouped with *D. nitedula* , while *D. laniger* was placed in the basal position to these species.

Based on *CYTB* data, *D. laniger* populations in Türkiye were clustered in two main haplogroups according to the results reported by Çetintaş et al. (2018), who suggested that the two haplogroups could be considered as distinct subspecies. Moreover, the authors noted the existence of geographical barriers between the two haplogroups. Later, Çetintaş et al. (2022) used mitochondrial and nuclear DNA sequences and reported a genetic distance of 7% between the Eastern and Western lineages of *D. laniger* . According to Çetintaş et al. (2022), the *D. laniger* lineages were monophyletic, and the easternmost distributional border of the Western lineage was likely the Saimbeyli—Tufanbeyli line, whereas the westernmost distributional border of the Eastern lineage was the Tahtalı Mountains on the provincial borders of Adana and Kahramanmaraş, Türkiye. Based on both morphological and genetic differences, Çetintaş et al. (2022) suggested that the Eastern lineage of *D. laniger* be considered as a new species within the genus *Dryomys*.

In the present study, *CYTB* sequences of *D. laniger* were grouped into two main lineages, as found in previous studies (Çetintaş et al. 2018, 2022; Kankılıç et al. 2018), comprising Eastern and Western lineages, which were deeply divergent. The Eastern lineage included samples from Kahramanmaraş and Tunceli, whereas the Western lineage contained samples from Niğde and Antalya. Moreover, in a Bayesian analysis based on mitochondrial *ND1* sequences, Kankılıç et al. (2018) included *D. laniger* haplotypes from Adana and Niğde, which were grouped into the Western lineage, as suggested by Çetintaş et al. (2022). In the current study, the mean genetic distance between the Eastern and the Western lineages was found to be 7%, similar to that reported by Çetintaş et al. (2022). In addition to the two main lineages within *D. laniger* , the phylogenetic analyses revealed that the Western lineage of *D. laniger* comprised two sublineages: W Laniger_1 contained samples from Antalya and W Laniger_2 included samples from Niğde, which might be an indication of subspecific variation, although data from the nuclear genome would be required to further evaluate this hypothesis.

In the Iranian Plateau and adjacent areas, phylogenetic relationships among subpopulations of *D. nitedula* were re‐evaluated by Mohammadi et al. (2021), who used mitochondrial (*CYTB*) and nuclear (*IRBP*) DNA markers. Analyses revealed four main lineages and multiple sublineages: (1) the Kopet Dagh–Zagros lineage, which included the Kopet Dagh and Zagros sublineages; (2) the Caucasian lineage containing the Lesser Caucasus and Alborz, central and eastern Great Caucasus, and western Great Caucasus and Russian sublineages; (3) the European–Apennine lineage containing the Eastern European, Calabrian, and Eastern Alps sublineages; and (4) the central Iranian Plateau lineage. Moreover, Mohammadi et al. (2021) confirmed that the Central Iranian Plateau lineage was a distinct lineage with an independent evolutionary trajectory, and they emphasized that *D. nitedula* likely represented a complex comprising several distinct species due to high genetic distance values and the inferred phylogenetic relationships observed among the main lineages. In a study investigating the evolutionary history of *D. nitedula* based on *CYTB* sequences of samples from Italy, Hungary, Lithuania, the Czech Republic, Russia, Bulgaria, Romania, Austria, and Greece, phylogenetic analyses revealed the existence of five main lineages, two of which were highly distinct from the Iranian and the Russian regions (El Mojahid et al. 2022).

In the current study, the BI and ML analyses revealed the presence of eight main lineages within *D. nitedula* (Figure 4, File S8). Three of these lineages (Eastern Europe, Central Caucasus, and Western Caucasus) were named by Grigoryeva et al. (2015), whereas seven of them (Kopet Dagh, Zagros, Lesser Caucasus and Alborz, Central and Eastern Great Caucasus, Western Great Caucasus, Eastern Europe, and Central Iran) were named by Mohammadi et al. (2021). The eighth lineage, Anatolia, is newly named based on the results of our study. The Turkish samples of *D. nitedula* were clustered into two main lineages: (i) Lesser Caucasus and Alborz included samples from Georgia, Iran and Türkiye, and (ii) Anatolia comprised samples from Türkiye. When considering the BI and ML tree topologies and the K2P pairwise genetic distances based on the *CYTB* sequences, the eight main lineages were quite divergent from each other (genetic distances ranged from 6% to 13.6%) and we also observed sublineages that were also deeply divergent within most of the *D. nitedula* lineages. In this context, our study confirmed the results of both Grigoryeva et al. (2015) and Mohammadi et al. (2021).

### 4.4 Some Insights for Intraspecific Relationships within *D. nitedula* and *D. laniger*

Many mammal species inhabiting the Anatolian part of Türkiye have relatively high levels of intraspecific genetic variation (Gündüz et al. 2005, 2007, 2023; Gür 2013; İbiş et al. 2014, 2018, 2023; Kankılıç et al. 2018, 2019; Arslan et al. 2020) due to climatic and topographic factors affecting the populations. Notably, this region includes a contact zone for three known biodiversity hotspots: the Caucasus, the Iran–Anatolian and the Mediterranean (Mittermeier et al. 2005; Bilgin 2011; Şekercioğlu et al. 2011). Anatolian animal populations have likely differentiated in response to phylogeographic barriers associated with these three different hotspot regions, leading to the evolution of major clades or lineages in the Anatolian region of Türkiye (Bilgin 2011).

As shown in the phylogenetic trees (Figure 4, File S8), both *D. nitedula* and *D. laniger* lineages were split into deeply divergent lineages and sublineages corresponding to phylogeographic breaks, which are often considered a consequence of long‐standing barriers to gene flow and attributed to both ecological and historical factors (Ye et al. 2017). The current study suggests that two phylogeographic breaks from the west to the east for *D. laniger* have occurred, including the Seyhan River that might be a barrier between the two main *CYTB* lineages (Eastern and Western), and the Göksu River that might be a barrier between the two sublineages within the Western lineage. The deep divergence between the Eastern and the Western lineages observed in the trees was also supported by the 7% genetic distance value (Table 2). Considering the areas covered by the *D. nitedula* lineages, phylogeographic breaks have also occurred throughout the distribution area of this species, involving multiple possible barriers: (i) the Caucasian Mountains between the Lesser Caucasus and Alborz lineage and the Central and Eastern Great Caucasus lineage; (ii) the Kuban River between the Central and Eastern Great Caucasus lineage and the Western Great Caucasus lineage; (iii) the Çoruh River (or Yalnizçam Mountains, Mescit Mountains and Otlukbeli Mountains in the south‐southeast of the river) plus the Karasu River + Esence Mountains (in the north and west of Erzurum) between the Lesser Caucasus and Alborz lineage and the Anatolia lineage; (iv) the Zagros Mountains plus the Sefid Rud River between the Lesser Caucasus and Alborz lineage and the Zagros lineage; (v) the Zagros Mountains between the Zagros lineage and the Central Iran lineage; and (vi) the Don River plus the Volgo River between the Western Great Caucasus lineage and the European lineage. The lowest mean distance value of 6% was found between the Kopet Dag lineage and the Zagros lineage.

The distance values of the *CYTB* sequences between the lineages of *D. laniger* and *D. nitedula* were each within the inter‐specific genetic distance limits determined in mammals, from 2.50% to 19.23% (Bradley and Baker 2001). It was also emphasized by Baker and Bradley (2006) that when mammalian phylogroups were separated by > 5% genetic distance using the *CYTB* gene, each phylogroup may potentially represent a separate species. In this context, the present study confirmed the presence of potential new cryptic species and subspecies within both *D. laniger* and *D. nitedula* as pointed out in previous studies (Mohammadi et al. 2021; Çetintaş et al. 2018, 2022). The results of the current study propose the possibility of distinct and endemic species occurring in the Anatolian part of Türkiye for both *D. laniger* and *D. nitedula* . Similarly, İbiş et al. (2023) pointed out the existence of undescribed taxa within the *Crocidura* populations distributed in Iran and Türkiye based on the K2P distances of the *CYTB* gene. Gündüz et al. (2023) determined a K2P distance value of 11.79% between the *Talpa davidiana* populations based on *CYTB* sequences, and they described a new species, *Talpa hakkariensis*, using mitochondrial *CYTB* data along with nuclear *BRCA2* data and geometric morphometrics.

Finally, species separation based solely on gene trees derived from mitochondrial DNA data, particularly the splitting of previously described species, was not recommended by Zachos et al. (2013), because this kind of data are assumed as imprecise in the absence of nuclear DNA data. Although the present study and the previous studies (Mohammadi et al. 2021; Çetintaş et al. 2022; El Mojahid et al. 2022) proposed that some lineages within both *D. laniger* and *D. nitedula* could be considered distinct species, the phylogenetic positions of these lineages should be further investigated using mitochondrial and nuclear data obtained from the distribution areas of the two species through next‐generation sequencing technologies and also geometric morphometrics.

## 5 Conclusions

The current study provided new insights into the evolutionary relationships of mitochondrial lineages within the families Gliridae and Sciuridae, and contributed new complete mitogenomes available for *Dryomys*, *Glis*, and *Spermophilus*, whose samples were collected from throughout Türkiye. The analyses of both the complete mitogenomes and the *CYTB* sequences revealed that each species of the Turkish *Dryomys* was divided into at least two major lineages, which might represent distinct species. Other major lineages within the range of *D. nitedula* might also belong to more than one species. To summarize, further molecular and geometric morphometrics studies including larger sample sizes and more comprehensive taxonomic coverage are needed to fully elucidate intra‐ and inter‐species relationships within Gliridae and Sciuridae.

## Author Contributions

**Osman İbiş:** conceptualization (equal), data curation (lead), formal analysis (lead), funding acquisition (lead), investigation (equal), methodology (equal), project administration (lead), resources (equal), software (equal), supervision (equal), validation (equal), visualization (equal), writing – original draft (equal), writing – review and editing (equal). **Ahmet Yesari Selçuk:** conceptualization (equal), formal analysis (equal), investigation (equal), resources (equal), writing – review and editing (equal). **Saffet Teber:** data curation (equal), formal analysis (equal), investigation (equal), methodology (equal), software (equal), visualization (equal), writing – review and editing (equal). **Mehmet Baran:** data curation (equal), formal analysis (equal), investigation (equal), methodology (equal), software (equal), validation (equal), visualization (equal), writing – review and editing (equal). **Klaus‐Peter Koepfli:** conceptualization (equal), supervision (equal), validation (equal), writing – original draft (equal), writing – review and editing (equal). **Haluk Kefelioğlu:** conceptualization (equal), investigation (equal), validation (equal), writing – review and editing (equal). **Coşkun Tez:** conceptualization (equal), formal analysis (equal), funding acquisition (equal), investigation (equal), resources (equal), supervision (equal), validation (equal), writing – original draft (equal), writing – review and editing (equal).

## Ethics Statement

Research ethics committee approval was provided for the capture of the animals, permission No. E‐21264211‐288.04‐10822834 from The Republic of Türkiye Ministry of Agriculture and Forestry, General Directorate of Nature Conservation and National Parks in regard to 23/061 numbered in conjunction with Erciyes University Local Ethics Committee for Animal Experiments.

## Conflicts of Interest

The authors declare no conflicts of interest.

## Supporting information


**File S1.** Mitogenomic and mitochondrial *CYTB* sequences obtained from the GenBank database of Sciuromorpha (Gliridae and Sciuridae) and three outgroup species (bolded) used in the phylogenetic analyses.

**File S2.** Mitogenome annotations for *Dryomys laniger* (DrLanTR1), *D. nitedula* (DrNitTR2 and DrNitTR9), *Glis glis* (GlGlisTR2), *Spermophilus citellus* (SpCitTR1), *S. taurensis* (SpTauTR1) and *S. xanthopyrmnus* (SpXanTR1) from Türkiye.

**File S3.** Nucleotide composition features of mitogenomes for *Dryomys laniger* (DrLanTR1), *D. nitedula* (DrNitTR2 and DrNitTR9), *Glis glis* (GlGlisTR2), *Spermophilus citellus* (SpCitTR1), *S. taurensis* (SpTauTR1) and *S. xanthopyrmnus* (SpXanTR1) from Türkiye.

**File S4.** Comparison of codon usage in protein‐coding genes of the mitogenomes of *Dryomys laniger* (DrLanTR1), *D. nitedula* (DrNitTR2 and DrNitTR9), *Glis glis* (GlGlisTR2), *Spermophilus citellus* (SpCitTR1), *S. taurensis* (SpTauTR1) and *S. xanthopyrmnus* (SpXanTR1) from Türkiye.

**File S5.** Secondary structure predictions for 22 tRNAs identified in mitogenomes of *Dryomys laniger* (DrLanTR1), *D. nitedula* (DrNitTR2 and DrNitTR9), *Glis glis* (GlGlisTR2), *Spermophilus citellus* (SpCitTR1), *S. taurensis* (SpTauTR1) and *S. xanthopyrmnus* (SpXanTR1) from Türkiye.

**File S6.** ML phylogenetic tree reconstructed using mitogenomes for Sciuromorpha (Gliridae and Sciuridae) based on the GTR+G+I model and 1000 bootstrap replicates.

**File S7.** Haplotypes of *CYTB* sequences obtained from the NCBI database and Türkiye, and summary statistics of sequence variation for *Dryomys laniger* and *D. nitedula* .

**File S8.** ML phylogenetic tree reconstructed using mitochondrial *CYTB* sequences for Gliridae based on the GTR+G+I model and 10,000 bootstrap replicates.

## Data Availability

The Turkish Sciuromorpha mitogenome sequencing data are available at NCBI‐GenBank: PQ533836–PQ533851.
