# Supplementary material for: Mitogenomic Analysis of Glirids (Gliridae) and Squirrels (Sciuridae) From Türkiye: Evolutionary and Taxonomic Implications Within the Suborder Sciuromorpha
Source: Ecol Evol. 2025 Feb 12;15(2):e70956. doi: 10.1002/ece3.70956 (PMC11821457; doi:10.1002/ece3.70956)
Supplement: Supplementary file 2 — File S2. Mitogenome annotations for Dryomys laniger (DrLanTR1), D. nitedula (DrNitTR2 and DrNitTR9), Glis glis (GlGlisTR2), Spermophilus citellus (SpCitTR1), S. taurensis (SpTauTR1) and S. xanthopyrmnus (SpXanTR1) from Türkiye. [file ECE3-15-e70956-s008.docx]

**Supplement file 2**. Mitogenome annotations for *Dryomys laniger* (DrLanTR1), *D. nitedula* (DrNitTR2 and DrNitTR9), *Glis glis* (GlGlisTR2), *Spermophilus citellus* (SpCitTR1), *S. taurensis* (SpTauTR1) and *S. xanthopyrmnus* (SpXanTR1) from Türkiye.

| ***Dryomys* *laniger*, 1899, DrLanTR1** | | | | | | | | | |
| --- | --- | --- | --- | --- | --- | --- | --- | --- | --- |
| Name | Type | S | Position |  | Size (bp) | Start | Stop | Anticodon | I.S |
| tRNA-Phe | tRNA | forward | 1 | 67 | 67 |  |  | GAA | 0 |
| 12S rRNA | rRNA | forward | 68 | 1033 | 966 |  |  |  | 0 |
| tRNA-Val | tRNA | forward | 1034 | 1099 | 66 |  |  | TAC | 0 |
| 16S rRNA | rRNA | forward | 1100 | 2672 | 1573 |  |  |  | 0 |
| tRNA-Leu | tRNA | forward | 2673 | 2747 | 75 |  |  | TAA | 2 |
| ND1 CDS | CDS | forward | 2750 | 3706 | 957 | ATG | TAA |  | -1 |
| tRNA-Ile | tRNA | forward | 3706 | 3774 | 69 |  |  | GAT | -3 |
| tRNA-Gln | tRNA | reverse | 3772 | 3845 | 74 |  |  | TTG | 4 |
| tRNA-Met | tRNA | forward | 3850 | 3918 | 69 |  |  | CAT | 0 |
| ND2 CDS | CDS | forward | 3919 | 4962 | 1044 | ATT | TAG |  | -2 |
| tRNA-Trp | tRNA | forward | 4961 | 5028 | 68 |  |  | TCA | 2 |
| tRNA-Ala | tRNA | reverse | 5031 | 5098 | 68 |  |  | TGC | 10 |
| tRNA-Asn | tRNA | reverse | 5109 | 5181 | 73 |  |  | GTT | 2 |
| oL | rep_origin | forward | 5184 | 5215 | 32 |  |  |  | -1 |
| tRNA-Cys | tRNA | reverse | 5215 | 5284 | 70 |  |  | GCA | 0 |
| tRNA-Tyr | tRNA | reverse | 5285 | 5349 | 65 |  |  | GTA | 7 |
| COX1 CDS | CDS | forward | 5357 | 6898 | 1542 | ATG | TAA |  | 1 |
| tRNA-Ser | tRNA | reverse | 6900 | 6969 | 70 |  |  | TGA | 7 |
| tRNA-Asp | tRNA | forward | 6977 | 7048 | 72 |  |  | GTC | 1 |
| COX2 CDS | CDS | forward | 7050 | 7736 | 687 | ATG | TAA |  | 0 |
| tRNA-Lys | tRNA | forward | 7737 | 7804 | 68 |  |  | TTT | 1 |
| ATP8 CDS | CDS | forward | 7806 | 7997 | 192 | ATG | TAA |  | -31 |
| ATP6 CDS | CDS | forward | 7967 | 8647 | 681 | ATG | TAA |  | -1 |
| COX3 CDS | CDS | forward | 8647 | 9430 | 784 | ATG | T-- |  | 0 |
| tRNA-Gly | tRNA | forward | 9431 | 9499 | 69 |  |  | TCC | 0 |
| ND3 CDS | CDS | forward | 9500 | 9847 | 348 | ATT | TAA |  | 4 |
| tRNA-Arg | tRNA | forward | 9852 | 9919 | 68 |  |  | TCG | 3 |
| ND4L CDS | CDS | forward | 9923 | 10219 | 297 | ATG | TAA |  | -7 |
| ND4 CDS | CDS | forward | 10213 | 11590 | 1378 | ATG | T-- |  | 0 |
| tRNA-His | tRNA | forward | 11591 | 11660 | 70 |  |  | GTG | 0 |
| tRNA-Ser | tRNA | forward | 11661 | 11722 | 62 |  |  | GCT | 0 |
| tRNA-Leu | tRNA | forward | 11723 | 11792 | 70 |  |  | TAG | 0 |
| ND5 CDS | CDS | forward | 11793 | 13601 | 1809 | ATA | TAA |  | -4 |
| ND6 CDS | CDS | reverse | 13598 | 14122 | 525 | ATG | TAA |  | 0 |
| tRNA-Glu | tRNA | reverse | 14123 | 14191 | 69 |  |  | TTC | 5 |
| CYTB CDS | CDS | forward | 14197 | 15336 | 1140 | ATG | AGA |  | 0 |
| tRNA-Thr | tRNA | forward | 15337 | 15407 | 71 |  |  | TGT | 6 |
| tRNA-Pro | tRNA | reverse | 15414 | 15483 | 70 |  |  | TGG | 0 |
| D-loop | D-loop | forward | 15484 | 16629 | 1146 |  |  |  | 0 |

| ***Dryomys nitedula*,754, DrNitTR2** | | | | | | | | | |
| --- | --- | --- | --- | --- | --- | --- | --- | --- | --- |
| Name | Type | S | Position |  | Size (bp) | Start | Stop | Anticodon | I.S |
| tRNA-Phe | tRNA | forward | 1 | 66 | 66 |  |  | GAA | 0 |
| 12S rRNA | rRNA | forward | 67 | 1.035 | 969 |  |  |  | 0 |
| tRNA-Val | tRNA | forward | 1.036 | 1.101 | 66 |  |  | TAC | 0 |
| 16S rRNA | rRNA | forward | 1.102 | 2.671 | 1.570 |  |  |  | 0 |
| tRNA-Leu | tRNA | forward | 2.672 | 2.746 | 75 |  |  | TAA | 3 |
| ND1 CDS | CDS | forward | 2.750 | 3.706 | 957 | ATG | TAA |  | -1 |
| tRNA-Ile | tRNA | forward | 3.706 | 3.774 | 69 |  |  | GAT | -3 |
| tRNA-Gln | tRNA | reverse | 3.772 | 3.845 | 74 |  |  | TTG | 5 |
| tRNA-Met | tRNA | forward | 3.851 | 3.919 | 69 |  |  | CAT | 0 |
| ND2 CDS | CDS | forward | 3.920 | 4.963 | 1.044 | ATC | TAG |  | -2 |
| tRNA-Trp | tRNA | forward | 4.962 | 5.028 | 67 |  |  | TCA | 2 |
| tRNA-Ala | tRNA | reverse | 5.031 | 5.098 | 68 |  |  | TGC | 6 |
| tRNA-Asn | tRNA | reverse | 5.105 | 5.177 | 73 |  |  | GTT | 3 |
| oL | rep_origin | forward | 5.181 | 5.211 | 31 |  |  |  | -1 |
| tRNA-Cys | tRNA | reverse | 5.211 | 5.280 | 70 |  |  | GCA | 0 |
| tRNA-Tyr | tRNA | reverse | 5.281 | 5.345 | 65 |  |  | GTA | 9 |
| COX1 CDS | CDS | forward | 5.355 | 6.896 | 1.542 | ATG | TAA |  | 1 |
| tRNA-Ser | tRNA | reverse | 6.898 | 6.967 | 70 |  |  | TGA | 11 |
| tRNA-Asp | tRNA | forward | 6.979 | 7.049 | 71 |  |  | GTC | 1 |
| COX2 CDS | CDS | forward | 7.051 | 7.734 | 684 | ATG | TAA |  | 3 |
| tRNA-Lys | tRNA | forward | 7.738 | 7.806 | 69 |  |  | TTT | 1 |
| ATP8 CDS | CDS | forward | 7.808 | 7.999 | 192 | ATG | TAA |  | -31 |
| ATP6 CDS | CDS | forward | 7.969 | 8.649 | 681 | ATG | TAA |  | -1 |
| COX3 CDS | CDS | forward | 8.649 | 9.432 | 784 | ATG | T-- |  | 0 |
| tRNA-Gly | tRNA | forward | 9.433 | 9.502 | 70 |  |  | TCC | 0 |
| ND3 CDS | CDS | forward | 9.503 | 9.850 | 348 | ATT | TAA |  | 3 |
| tRNA-Arg | tRNA | forward | 9.854 | 9.922 | 69 |  |  | TCG | 1 |
| ND4L CDS | CDS | forward | 9.924 | 10.220 | 297 | ATG | TAA |  | -7 |
| ND4 CDS | CDS | forward | 10.214 | 11.591 | 1.378 | ATG | T-- |  | 0 |
| tRNA-His | tRNA | forward | 11.592 | 11.660 | 69 |  |  | GTG | 0 |
| tRNA-Ser | tRNA | forward | 11.661 | 11.722 | 62 |  |  | GCT | 0 |
| tRNA-Leu | tRNA | forward | 11.723 | 11.792 | 70 |  |  | TAG | 0 |
| ND5 CDS | CDS | forward | 11.793 | 13.598 | 1.806 | ATA | TAA |  | -4 |
| ND6 CDS | CDS | reverse | 13.595 | 14.119 | 525 | ATG | TAA |  | 0 |
| tRNA-Glu | tRNA | reverse | 14.120 | 14.188 | 69 |  |  | TTC | 5 |
| CYTB CDS | CDS | forward | 14.194 | 15.333 | 1.140 | ATG | AGA |  | 0 |
| tRNA-Thr | tRNA | forward | 15.334 | 15.403 | 70 |  |  | TGT | 7 |
| tRNA-Pro | tRNA | reverse | 15.411 | 15.478 | 68 |  |  | TGG | 0 |
| D-loop | D-loop | forward | 15.479 | 16.618 | 1.140 |  |  |  | 0 |

| ***Dryomys nitedula*, 1878, DrNitTR9** | | | | | | | | | |
| --- | --- | --- | --- | --- | --- | --- | --- | --- | --- |
| Name | Type | S | Position |  | Size (bp) | Start | Stop | Anticodon | I.S |
| tRNA-Phe | tRNA | forward | 1 | 67 | 67 |  |  | GAA | 0 |
| 12S rRNA | rRNA | forward | 68 | 1.035 | 968 |  |  |  | 0 |
| tRNA-Val | tRNA | forward | 1.036 | 1.101 | 66 |  |  | TAC | 0 |
| 16S rRNA | rRNA | forward | 1.102 | 2.671 | 1.570 |  |  |  | 0 |
| tRNA-Leu | tRNA | forward | 2.672 | 2.746 | 75 |  |  | TAA | 2 |
| ND1 CDS | CDS | forward | 2.749 | 3.705 | 957 | ATG | TAA |  | -1 |
| tRNA-Ile | tRNA | forward | 3.705 | 3.773 | 69 |  |  | GAT | -3 |
| tRNA-Gln | tRNA | reverse | 3.771 | 3.844 | 74 |  |  | TTG | 6 |
| tRNA-Met | tRNA | forward | 3.851 | 3.919 | 69 |  |  | CAT | 0 |
| ND2 CDS | CDS | forward | 3.920 | 4.963 | 1.044 | ATT | TAG |  | -2 |
| tRNA-Trp | tRNA | forward | 4.962 | 5.028 | 67 |  |  | TCA | 2 |
| tRNA-Ala | tRNA | reverse | 5.031 | 5.098 | 68 |  |  | TGC | 6 |
| tRNA-Asn | tRNA | reverse | 5.105 | 5.177 | 73 |  |  | GTT | 3 |
| oL | rep_origin | forward | 5.181 | 5.211 | 31 |  |  |  | -1 |
| tRNA-Cys | tRNA | reverse | 5.211 | 5.280 | 70 |  |  | GCA | 0 |
| tRNA-Tyr | tRNA | reverse | 5.281 | 5.345 | 65 |  |  | GTA | 9 |
| COX1 CDS | CDS | forward | 5.355 | 6.896 | 1.542 | ATG | TAA |  | 1 |
| tRNA-Ser | tRNA | reverse | 6.898 | 6.967 | 70 |  |  | TGA | 12 |
| tRNA-Asp | tRNA | forward | 6.980 | 7.050 | 71 |  |  | GTC | 1 |
| COX2 CDS | CDS | forward | 7.052 | 7.735 | 684 | ATG | TAA |  | 3 |
| tRNA-Lys | tRNA | forward | 7.739 | 7.806 | 68 |  |  | TTT | 1 |
| ATP8 CDS | CDS | forward | 7.808 | 7.999 | 192 | ATG | TAA |  | -31 |
| ATP6 CDS | CDS | forward | 7.969 | 8.649 | 681 | ATG | TAA |  | -1 |
| COX3 CDS | CDS | forward | 8.649 | 9.432 | 784 | ATG | T-- |  | 0 |
| tRNA-Gly | tRNA | forward | 9.433 | 9.502 | 70 |  |  | TCC | 0 |
| ND3 CDS | CDS | forward | 9.503 | 9.850 | 348 | ATA | TAG |  | 3 |
| tRNA-Arg | tRNA | forward | 9.854 | 9.922 | 69 |  |  | TCG | 1 |
| ND4L CDS | CDS | forward | 9.924 | 10.220 | 297 | ATG | TAA |  | -7 |
| ND4 CDS | CDS | forward | 10.214 | 11.591 | 1.378 | ATG | T-- |  | 0 |
| tRNA-His | tRNA | forward | 11.592 | 11.660 | 69 |  |  | GTG | 0 |
| tRNA-Ser | tRNA | forward | 11.661 | 11.722 | 62 |  |  | GCT | 0 |
| tRNA-Leu | tRNA | forward | 11.723 | 11.792 | 70 |  |  | TAG | 0 |
| ND5 CDS | CDS | forward | 11.793 | 13.598 | 1.806 | ATA | TAA |  | -4 |
| ND6 CDS | CDS | reverse | 13.595 | 14.119 | 525 | ATG | TAA |  | 0 |
| tRNA-Glu | tRNA | reverse | 14.120 | 14.188 | 69 |  |  | TTC | 5 |
| CYTB CDS | CDS | forward | 14.194 | 15.333 | 1.140 | ATG | AGA |  | 0 |
| tRNA-Thr | tRNA | forward | 15.334 | 15.403 | 70 |  |  | TGT | 7 |
| tRNA-Pro | tRNA | reverse | 15.411 | 15.478 | 68 |  |  | TGG | 0 |
| D-loop | D-loop | forward | 15.479 | 16.614 | 1.136 |  |  |  | 0 |

| ***Glis glis*, 1832, GlGlis2** | | | | | | | | | |
| --- | --- | --- | --- | --- | --- | --- | --- | --- | --- |
| Name | Type | S | Position |  | Size (bp) | Start | Stop | Anticodon | I.S |
| tRNA-Phe | tRNA | forward | 1 | 69 | 69 |  |  | GAA | 0 |
| 12S rRNA | rRNA | forward | 70 | 1.024 | 955 |  |  |  | 0 |
| tRNA-Val | tRNA | forward | 1.025 | 1.090 | 66 |  |  | TAC | 0 |
| 16S rRNA | rRNA | forward | 1.091 | 2.648 | 1.558 |  |  |  | 0 |
| tRNA-Leu | tRNA | forward | 2.649 | 2.723 | 75 |  |  | TAA | 2 |
| ND1 CDS | CDS | forward | 2.726 | 3.682 | 957 | ATG | TAG |  | -2 |
| tRNA-Ile | tRNA | forward | 3.681 | 3.749 | 69 |  |  | GAT | -3 |
| tRNA-Gln | tRNA | reverse | 3.747 | 3.820 | 74 |  |  | TTG | 4 |
| tRNA-Met | tRNA | forward | 3.825 | 3.893 | 69 |  |  | CAT | 0 |
| ND2 CDS | CDS | forward | 3.894 | 4.937 | 1.044 | ATC | TAG |  | -2 |
| tRNA-Trp | tRNA | forward | 4.936 | 5.003 | 68 |  |  | TCA | 4 |
| tRNA-Ala | tRNA | reverse | 5.008 | 5.075 | 68 |  |  | TGC | 12 |
| tRNA-Asn | tRNA | reverse | 5.088 | 5.160 | 73 |  |  | GTT | 2 |
| oL | rep_origin | forward | 5.163 | 5.192 | 30 |  |  |  | -1 |
| tRNA-Cys | tRNA | reverse | 5.192 | 5.259 | 68 |  |  | GCA | 0 |
| tRNA-Tyr | tRNA | reverse | 5.260 | 5.324 | 65 |  |  | GTA | 8 |
| COX1 CDS | CDS | forward | 5.333 | 6.874 | 1.542 | ATG | TAG |  | 3 |
| tRNA-Ser | tRNA | reverse | 6.878 | 6.947 | 70 |  |  | TGA | 10 |
| tRNA-Asp | tRNA | forward | 6.958 | 7.026 | 69 |  |  | GTC | 1 |
| COX2 CDS | CDS | forward | 7.028 | 7.711 | 684 | ATG | TAA |  | 3 |
| tRNA-Lys | tRNA | forward | 7.715 | 7.783 | 69 |  |  | TTT | 1 |
| ATP8 CDS | CDS | forward | 7.785 | 7.976 | 192 | ATG | TAA |  | -31 |
| ATP6 CDS | CDS | forward | 7.946 | 8.626 | 681 | ATG | TAA |  | -1 |
| COX3 CDS | CDS | forward | 8.626 | 9.409 | 784 | ATG | T-- |  | 0 |
| tRNA-Gly | tRNA | forward | 9.410 | 9.477 | 68 |  |  | TCC | 0 |
| ND3 CDS | CDS | forward | 9.478 | 9.824 | 347 | ATT | TA- |  | 0 |
| tRNA-Arg | tRNA | forward | 9.825 | 9.894 | 70 |  |  | TCG | 1 |
| ND4L CDS | CDS | forward | 9.896 | 10.192 | 297 | ATG | TAA |  | -7 |
| ND4 CDS | CDS | forward | 10.186 | 11.563 | 1.378 | ATG | T-- |  | 0 |
| tRNA-His | tRNA | forward | 11.564 | 11.634 | 71 |  |  | GTG | 0 |
| tRNA-Ser | tRNA | forward | 11.635 | 11.695 | 61 |  |  | GCT | 0 |
| tRNA-Leu | tRNA | forward | 11.696 | 11.765 | 70 |  |  | TAG | 0 |
| ND5 CDS | CDS | forward | 11.766 | 13.574 | 1.809 | ATA | TAA |  | -4 |
| ND6 CDS | CDS | reverse | 13.571 | 14.095 | 525 | ATG | TAG |  | 0 |
| tRNA-Glu | tRNA | reverse | 14.096 | 14.164 | 69 |  |  | TTC | 4 |
| CYTB CDS | CDS | forward | 14.169 | 15.308 | 1.140 | ATG | AGA |  | 0 |
| tRNA-Thr | tRNA | forward | 15.309 | 15.376 | 68 |  |  | TGT | 1 |
| tRNA-Pro | tRNA | reverse | 15.378 | 15.443 | 66 |  |  | TGG | 0 |
| D-loop | D-loop | forward | 15.444 | 16.601 | 1.158 |  |  |  | 0 |

| **Spermophilus citellus, 336, SpCit1** | | | | | | | | | |
| --- | --- | --- | --- | --- | --- | --- | --- | --- | --- |
| Name | Type | S | Position |  | Size (bp) | Start | Stop | Anticodon | I.S |
| tRNA-Phe | tRNA | forward | 1 | 70 | 70 |  |  | GAA | 0 |
| 12S rRNA | rRNA | forward | 71 | 1.044 | 974 |  |  |  | 0 |
| tRNA-Val | tRNA | forward | 1.045 | 1.114 | 70 |  |  | TAC | 0 |
| 16S rRNA | rRNA | forward | 1.115 | 2.678 | 1.564 |  |  |  | 0 |
| tRNA-Leu | tRNA | forward | 2.679 | 2.752 | 74 |  |  | TAA | 3 |
| ND1 CDS | CDS | forward | 2.756 | 3.712 | 957 | ATG | TAA |  | -1 |
| tRNA-Ile | tRNA | forward | 3.712 | 3.780 | 69 |  |  | GAT | -3 |
| tRNA-Gln | tRNA | reverse | 3.778 | 3.849 | 72 |  |  | TTG | 7 |
| tRNA-Met | tRNA | forward | 3.857 | 3.925 | 69 |  |  | CAT | 0 |
| ND2 CDS | CDS | forward | 3.926 | 4.969 | 1.044 | ATT | TAG |  | -2 |
| tRNA-Trp | tRNA | forward | 4.968 | 5.035 | 68 |  |  | TCA | 3 |
| tRNA-Ala | tRNA | reverse | 5.039 | 5.107 | 69 |  |  | TGC | 6 |
| tRNA-Asn | tRNA | reverse | 5.114 | 5.186 | 73 |  |  | GTT | 2 |
| oL | rep_origin | forward | 5.189 | 5.218 | 30 |  |  |  | -1 |
| tRNA-Cys | tRNA | reverse | 5.218 | 5.284 | 67 |  |  | GCA | 0 |
| tRNA-Tyr | tRNA | reverse | 5.285 | 5.350 | 66 |  |  | GTA | 8 |
| COX1 CDS | CDS | forward | 5.359 | 6.900 | 1.542 | ATG | TAA |  | 2 |
| tRNA-Ser | tRNA | reverse | 6.903 | 6.971 | 69 |  |  | TGA | 3 |
| tRNA-Asp | tRNA | forward | 6.975 | 7.043 | 69 |  |  | GTC | 0 |
| COX2 CDS | CDS | forward | 7.044 | 7.727 | 684 | ATG | TAA |  | 3 |
| tRNA-Lys | tRNA | forward | 7.731 | 7.797 | 67 |  |  | TTT | 1 |
| ATP8 CDS | CDS | forward | 7.799 | 8.002 | 204 | ATG | TAA |  | -43 |
| ATP6 CDS | CDS | forward | 7.960 | 8.640 | 681 | ATG | TAA |  | -1 |
| COX3 CDS | CDS | forward | 8.640 | 9.423 | 784 | ATG | T-- |  | 0 |
| tRNA-Gly | tRNA | forward | 9.424 | 9.493 | 70 |  |  | TCC | 0 |
| ND3 CDS | CDS | forward | 9.494 | 9.840 | 347 | ATA | TA- |  | 0 |
| tRNA-Arg | tRNA | forward | 9.841 | 9.907 | 67 |  |  | TCG | 1 |
| ND4L CDS | CDS | forward | 9.909 | 10.205 | 297 | ATG | TAA |  | -7 |
| ND4 CDS | CDS | forward | 10.199 | 11.576 | 1.378 | ATG | T-- |  | 0 |
| tRNA-His | tRNA | forward | 11.577 | 11.645 | 69 |  |  | GTG | 0 |
| tRNA-Ser | tRNA | forward | 11.646 | 11.704 | 59 |  |  | GCT | 0 |
| tRNA-Leu | tRNA | forward | 11.705 | 11.774 | 70 |  |  | TAG | 0 |
| ND5 CDS | CDS | forward | 11.775 | 13.592 | 1.818 | ATT | TAA |  | -17 |
| ND6 CDS | CDS | reverse | 13.576 | 14.100 | 525 | ATG | AGA |  | 0 |
| tRNA-Glu | tRNA | reverse | 14.101 | 14.169 | 69 |  |  | TTC | 4 |
| CYTB CDS | CDS | forward | 14.174 | 15.313 | 1.140 | ATG | AGA |  | 0 |
| tRNA-Thr | tRNA | forward | 15.314 | 15.379 | 66 |  |  | TGT | 3 |
| tRNA-Pro | tRNA | reverse | 15.383 | 15.449 | 67 |  |  | TGG | 0 |
| D-loop | D-loop | forward | 15.450 | 16.449 | 1.000 |  |  |  | 0 |

| **S*permophilus taurensi*, 339, SpTau1** | | | | | | | | | |
| --- | --- | --- | --- | --- | --- | --- | --- | --- | --- |
| Name | Type | S | Position |  | Size (bp) | Start | Stop | Anticodon | I.S |
| tRNA-Phe | tRNA | forward | 1 | 70 | 70 |  |  | GAA | 0 |
| 12S rRNA | rRNA | forward | 71 | 1.041 | 971 |  |  |  | 0 |
| tRNA-Val | tRNA | forward | 1.042 | 1.110 | 69 |  |  | TAC | 0 |
| 16S rRNA | rRNA | forward | 1.111 | 2.676 | 1.566 |  |  |  | 0 |
| tRNA-Leu | tRNA | forward | 2.677 | 2.750 | 74 |  |  | TAA | 3 |
| ND1 CDS | CDS | forward | 2.754 | 3.710 | 957 | ATG | TAA |  | -1 |
| tRNA-Ile | tRNA | forward | 3.710 | 3.778 | 69 |  |  | GAT | -3 |
| tRNA-Gln | tRNA | reverse | 3.776 | 3.851 | 76 |  |  | TTG | 3 |
| tRNA-Met | tRNA | forward | 3.855 | 3.923 | 69 |  |  | CAT | 0 |
| ND2 CDS | CDS | forward | 3.924 | 4.967 | 1.044 | ATT | TAG |  | -2 |
| tRNA-Trp | tRNA | forward | 4.966 | 5.033 | 68 |  |  | TCA | 3 |
| tRNA-Ala | tRNA | reverse | 5.037 | 5.105 | 69 |  |  | TGC | 5 |
| tRNA-Asn | tRNA | reverse | 5.111 | 5.183 | 73 |  |  | GTT | 2 |
| oL | rep_origin | forward | 5.186 | 5.215 | 30 |  |  |  | -1 |
| tRNA-Cys | tRNA | reverse | 5.215 | 5.281 | 67 |  |  | GCA | 0 |
| tRNA-Tyr | tRNA | reverse | 5.282 | 5.347 | 66 |  |  | GTA | 8 |
| COX1 CDS | CDS | forward | 5.356 | 6.897 | 1.542 | ATG | TAA |  | 2 |
| tRNA-Ser | tRNA | reverse | 6.900 | 6.968 | 69 |  |  | TGA | 3 |
| tRNA-Asp | tRNA | forward | 6.972 | 7.040 | 69 |  |  | GTC | 0 |
| COX2 CDS | CDS | forward | 7.041 | 7.724 | 684 | ATG | TAA |  | 3 |
| tRNA-Lys | tRNA | forward | 7.728 | 7.794 | 67 |  |  | TTT | 1 |
| ATP8 CDS | CDS | forward | 7.796 | 7.999 | 204 | ATG | TAA |  | -43 |
| ATP6 CDS | CDS | forward | 7.957 | 8.637 | 681 | ATG | TAA |  | -1 |
| COX3 CDS | CDS | forward | 8.637 | 9.420 | 784 | ATG | T-- |  | 0 |
| tRNA-Gly | tRNA | forward | 9.421 | 9.490 | 70 |  |  | TCC | 0 |
| ND3 CDS | CDS | forward | 9.491 | 9.837 | 347 | ATA | TA- |  | 0 |
| tRNA-Arg | tRNA | forward | 9.838 | 9.904 | 67 |  |  | TCG | 1 |
| ND4L CDS | CDS | forward | 9.906 | 10.202 | 297 | ATG | TAA |  | -7 |
| ND4 CDS | CDS | forward | 10.196 | 11.573 | 1.378 | ATG | T-- |  | 0 |
| tRNA-His | tRNA | forward | 11.574 | 11.642 | 69 |  |  | GTG | 0 |
| tRNA-Ser | tRNA | forward | 11.643 | 11.701 | 59 |  |  | GCT | 0 |
| tRNA-Leu | tRNA | forward | 11.702 | 11.771 | 70 |  |  | TAG | 0 |
| ND5 CDS | CDS | forward | 11.772 | 13.589 | 1.818 | ATT | TAA |  | -17 |
| ND6 CDS | CDS | reverse | 13.573 | 14.097 | 525 | ATG | AGA |  | 0 |
| tRNA-Glu | tRNA | reverse | 14.098 | 14.166 | 69 |  |  | TTC | 4 |
| CYTB CDS | CDS | forward | 14.171 | 15.310 | 1.140 | ATG | AGA |  | 0 |
| tRNA-Thr | tRNA | forward | 15.311 | 15.376 | 66 |  |  | TGT | 3 |
| tRNA-Pro | tRNA | reverse | 15.380 | 15.446 | 67 |  |  | TGG | 0 |
| D-loop | D-loop | forward | 15.447 | 16.447 | 1.001 |  |  |  | 0 |

| ***Spermophilus xanthopyrmnus*, 250, SpXan1** | | | | | | | | | |
| --- | --- | --- | --- | --- | --- | --- | --- | --- | --- |
| Name | Type | S | Position |  | Size (bp) | Start | Stop | Anticodon | I.S |
| tRNA-Phe | tRNA | forward | 1 | 70 | 70 |  |  | GAA | 0 |
| 12S rRNA | rRNA | forward | 71 | 1.055 | 985 |  |  |  | 0 |
| tRNA-Val | tRNA | forward | 1.056 | 1.125 | 70 |  |  | TAC | 0 |
| 16S rRNA | rRNA | forward | 1.126 | 2.691 | 1.566 |  |  |  | 0 |
| tRNA-Leu | tRNA | forward | 2.692 | 2.765 | 74 |  |  | TAA | 3 |
| ND1 CDS | CDS | forward | 2.769 | 3.725 | 957 | ATG | TAA |  | -1 |
| tRNA-Ile | tRNA | forward | 3.725 | 3.793 | 69 |  |  | GAT | -3 |
| tRNA-Gln | tRNA | reverse | 3.791 | 3.862 | 72 |  |  | TTG | 7 |
| tRNA-Met | tRNA | forward | 3.870 | 3.938 | 69 |  |  | CAT | 0 |
| ND2 CDS | CDS | forward | 3.939 | 4.982 | 1.044 | ATT | TAG |  | -2 |
| tRNA-Trp | tRNA | forward | 4.981 | 5.048 | 68 |  |  | TCA | 2 |
| tRNA-Ala | tRNA | reverse | 5.051 | 5.119 | 69 |  |  | TGC | 5 |
| tRNA-Asn | tRNA | reverse | 5.125 | 5.197 | 73 |  |  | GTT | 2 |
| oL | rep_origin | forward | 5.200 | 5.229 | 30 |  |  |  | -1 |
| tRNA-Cys | tRNA | reverse | 5.229 | 5.295 | 67 |  |  | GCA | 0 |
| tRNA-Tyr | tRNA | reverse | 5.296 | 5.361 | 66 |  |  | GTA | 8 |
| COX1 CDS | CDS | forward | 5.370 | 6.911 | 1.542 | ATG | TAA |  | 2 |
| tRNA-Ser | tRNA | reverse | 6.914 | 6.982 | 69 |  |  | TGA | 3 |
| tRNA-Asp | tRNA | forward | 6.986 | 7.054 | 69 |  |  | GTC | 0 |
| COX2 CDS | CDS | forward | 7.055 | 7.738 | 684 | ATG | TAA |  | 3 |
| tRNA-Lys | tRNA | forward | 7.742 | 7.808 | 67 |  |  | TTT | 1 |
| ATP8 CDS | CDS | forward | 7.810 | 8.016 | 207 | ATG | TAG |  | -46 |
| ATP6 CDS | CDS | forward | 7.971 | 8.651 | 681 | ATG | TAA |  | -1 |
| COX3 CDS | CDS | forward | 8.651 | 9.434 | 784 | ATG | T-- |  | 0 |
| tRNA-Gly | tRNA | forward | 9.435 | 9.505 | 71 |  |  | TCC | 0 |
| ND3 CDS | CDS | forward | 9.506 | 9.852 | 347 | ATT | TA- |  | 0 |
| tRNA-Arg | tRNA | forward | 9.853 | 9.919 | 67 |  |  | TCG | 1 |
| ND4L CDS | CDS | forward | 9.921 | 10.217 | 297 | ATG | TAA |  | -7 |
| ND4 CDS | CDS | forward | 10.211 | 11.588 | 1.378 | ATG | T-- |  | 0 |
| tRNA-His | tRNA | forward | 11.589 | 11.657 | 69 |  |  | GTG | 0 |
| tRNA-Ser | tRNA | forward | 11.658 | 11.716 | 59 |  |  | GCT | 0 |
| tRNA-Leu | tRNA | forward | 11.717 | 11.786 | 70 |  |  | TAG | 0 |
| ND5 CDS | CDS | forward | 11.787 | 13.604 | 1.818 | ATT | TAA |  | -17 |
| ND6 CDS | CDS | reverse | 13.588 | 14.112 | 525 | ATG | AGA |  | 0 |
| tRNA-Glu | tRNA | reverse | 14.113 | 14.181 | 69 |  |  | TTC | 4 |
| CYTB CDS | CDS | forward | 14.186 | 15.325 | 1.140 | ATG | AGA |  | 0 |
| tRNA-Thr | tRNA | forward | 15.326 | 15.390 | 65 |  |  | TGT | 3 |
| tRNA-Pro | tRNA | reverse | 15.394 | 15.460 | 67 |  |  | TGG | 0 |
| D-loop | D-loop | forward | 15.461 | 16.469 | 1.009 |  |  |  | 0 |
