# Supplementary material for: Mitogenomic Analysis of Glirids (Gliridae) and Squirrels (Sciuridae) From Türkiye: Evolutionary and Taxonomic Implications Within the Suborder Sciuromorpha
Source: Ecol Evol. 2025 Feb 12;15(2):e70956. doi: 10.1002/ece3.70956 (PMC11821457; doi:10.1002/ece3.70956)
Supplement: Supplementary file 3 — File S3. Nucleotide composition features of mitogenomes for Dryomys laniger (DrLanTR1), D. nitedula (DrNitTR2 and DrNitTR9), Glis glis (GlGlisTR2), Spermophilus citellus (SpCitTR1), S. taurensis (SpTauTR1) and S. xanthopyrmnus (SpXanTR1) from Türkiye. [file ECE3-15-e70956-s003.docx]

**Supplement file 3.** Nucleotide composition features of mitogenomes for *Dryomys laniger* (DrLanTR1), *D. nitedula* (DrNitTR2 and DrNitTR9), *Glis glis* (GlGlisTR2), *Spermophilus citellus* (SpCitTR1), *S. taurensis* (SpTauTR1) and *S. xanthopyrmnus* (SpXanTR1) from Türkiye.

|  |  | |  | |  |  |  |  | |  | |  | |  |  |  |  |  |  |  |
| --- | --- | --- | --- | --- | --- | --- | --- | --- | --- | --- | --- | --- | --- | --- | --- | --- | --- | --- | --- | --- |
|  | | **Species** | | **Common Name/Collection NO** | | | | | **Size (bp)** | | **% A** | | **% T** | | **% G** | **% C** | **% A + T** | **% G + C** | **AT-Skew** | **GC-Skew** |
| **Mitogenome** | | *D. nitedula* | | Forest dormouse / 754 | | | | | 16,618 | | 32,9 | | 28,1 | | 13,4 | 25,6 | 61,0 | 39,0 | 0,0782 | -0,3113 |
|  |  | *D. nitedula* | | Forest dormouse / 1878 | | | | | 16,614 | | 33,1 | | 27,8 | | 13,4 | 25,7 | 60,9 | 39,1 | 0,0863 | -0,3154 |
|  |  | *D. laniger* | | Woolly dormouse / 1899 | | | | | 16,629 | | 32,8 | | 29,9 | | 13,3 | 24 | 62,7 | 37,3 | 0,0455 | -0,2873 |
|  |  | *G. glis* | | Fat dormouse / 1832 | | | | | 16,601 | | 32,5 | | 31,6 | | 12,2 | 23,7 | 64,1 | 35,9 | 0,01474 | -0,3223 |
|  |  | *S. xanthoprymnus* | | Anatolian ground squirrel / 250 | | | | | 16,469 | | 32,6 | | 32,0 | | 12,4 | 23,0 | 64,6 | 35,4 | 0,0094 | -0,2998 |
|  |  | *S. citellus* | | European suslik / 336 | | | | | 16,449 | | 32,7 | | 31,9 | | 12,5 | 23,0 | 64,5 | 35,5 | 0,012 | -0,2976 |
|  |  | *S. taurensis* | | Taurus ground squirrel / 339 | | | | | 16,447 | | 32,7 | | 32,0 | | 12,4 | 23,0 | 64,6 | 35,4 | 0,011 | -0,2995 |
|  |  | *S. anomalus* | | Caucasian squirrel / 345 | | | | | 16,507 | | 31,5 | | 30,2 | | 13,1 | 25,2 | 61,7 | 38,3 | 0,022 | −0.320 |
|  |  | *S. vulgaris* | | Eurasian red squirrel / 1418 | | | | | 16,511 | | 32,1 | | 30,9 | | 12,5 | 24,5 | 63,0 | 37,0 | 0,020 | −0.323 |
| **PCGs** | | *D. nitedula* | | Forest dormouse / 754 | | | | | 11.377 | | 31,6 | | 28,9 | | 12,2 | 27,2 | 60,5 | 39,5 | 0,0452 | -0,3807 |
|  |  | *D. nitedula* | | Forest dormouse / 1878 | | | | | 11,378 | | 31,9 | | 28,6 | | 12,2 | 27,3 | 60,5 | 39,5 | 0,0544 | -0,3833 |
|  |  | *D. laniger* | | Woolly dormouse / 1899 | | | | | 11,384 | | 31,6 | | 30,8 | | 12,3 | 25,4 | 62,3 | 37,7 | 0,0124 | -0,3484 |
|  |  | *G. glis* | | Fat dormouse / 1832 | | | | | 11,380 | | 31,3 | | 32,9 | | 11,1 | 24,7 | 64,3 | 35,7 | -0,0246 | -0,3815 |
|  |  | *S. xanthoprymnus* | | Anatolian ground squirrel / 250 | | | | | 11,404 | | 31,4 | | 33,2 | | 11,3 | 24,2 | 64,6 | 35,4 | -0,02811 | -0,3645 |
|  |  | *S. citellus* | | European suslik / 336 | | | | | 11,401 | | 31,5 | | 33,1 | | 11,2 | 24,2 | 64,6 | 35,4 | -0,0255 | -0,3658 |
|  |  | *S. taurensis* | | Taurus ground squirrel / 339 | | | | | 11,401 | | 31,4 | | 33,2 | | 11,2 | 24,1 | 64,6 | 35,4 | -0,0282 | -0,3644 |
|  |  | *S. anomalus* | | Caucasian squirrel / 345 | | | | | 11,404 | | 30,4 | | 31,2 | | 11,8 | 26,6 | 61,6 | 38,4 | −0.013 | −0.385 |
|  |  | *S. vulgaris* | | Eurasian red squirrel / 1418 | | | | | 11,398 | | 31,0 | | 31,6 | | 11,4 | 26,0 | 62,6 | 37,4 | −0.012 | −0.391 |
| **tRNA genes** | | *D. nitedula* | | Forest dormouse / 754 | | | | | 1,519 | | 35,4 | | 28,6 | | 14,9 | 20,9 | 64,2 | 35,8 | 0,1076 | -0,1691 |
|  |  | *D. nitedula* | | Forest dormouse / 1878 | | | | | 1,519 | | 35,4 | | 28,9 | | 15,0 | 20,7 | 64,3 | 35,7 | 0,1013 | -0,1586 |
|  |  | *D. laniger* | | Woolly dormouse / 1899 | | | | | 1,523 | | 35,9 | | 30,3 | | 14,6 | 19,2 | 66,3 | 33,7 | 0,0842 | -0,1361 |
|  |  | *G. glis* | | Fat dormouse / 1832 | | | | | 1,515 | | 36,0 | | 30,8 | | 14,0 | 19,3 | 66,7 | 33,3 | 0,0781 | -0,1587 |
|  |  | *S. xanthoprymnus* | | Anatolian ground squirrel / 250 | | | | | 1,509 | | 35,2 | | 30,8 | | 14,7 | 19,3 | 66,0 | 34,0 | 0,0662 | -0,1345 |
|  |  | *S. citellus* | | European suslik / 336 | | | | | 1,509 | | 35,2 | | 30,2 | | 14,8 | 19,7 | 65,4 | 34,6 | 0,0759 | -0,1417 |
|  |  | *S. taurensis* | | Taurus ground squirrel / 339 | | | | | 1,512 | | 35,3 | | 30,6 | | 14,6 | 19,5 | 65,9 | 34,1 | 0,0722 | -0,1434 |
|  |  | *S. anomalus* | | Caucasian squirrel / 345 | | | | | 1,507 | | 35,5 | | 29,0 | | 14,9 | 20,6 | 64,5 | 35,5 | 0,101 | −0.159 |
|  |  | *S. vulgaris* | | Eurasian red squirrel / 1418 | | | | | 1,511 | | 35,8 | | 30,0 | | 14,5 | 19,7 | 65,8 | 34,2 | 0,087 | −0.151 |
| **rRNA genes** | | *D. nitedula* | | Forest dormouse / 754 | | | | | 2,539 | | 37,1 | | 23,9 | | 17,9 | 21,2 | 61,0 | 39,0 | 0,2157 | -0,0837 |
|  |  | *D. nitedula* | | Forest dormouse / 1878 | | | | | 2,538 | | 37,0 | | 23,1 | | 18,0 | 21,8 | 60,1 | 39,9 | 0,2306 | -0,0948 |
|  |  | *D. laniger* | | Woolly dormouse / 1899 | | | | | 2,539 | | 36,6 | | 25,5 | | 17,6 | 20,2 | 62,2 | 37,8 | 0,1787 | -0,0697 |
|  |  | *G. glis* | | Fat dormouse / 1832 | | | | | 2,513 | | 35,9 | | 26,3 | | 16,8 | 21,0 | 62,2 | 37,8 | 0,1554 | -0,1115 |
|  |  | *S. xanthoprymnus* | | Anatolian ground squirrel / 250 | | | | | 2,551 | | 37,0 | | 27,1 | | 16,2 | 19,7 | 64,1 | 35,9 | 0,1535 | -0,0982 |
|  |  | *S. citellus* | | European suslik / 336 | | | | | 2,538 | | 36,8 | | 27,0 | | 16,6 | 19,6 | 63,8 | 36,2 | 0,1525 | -0,0816 |
|  |  | *S. taurensis* | | Taurus ground squirrel / 339 | | | | | 2,537 | | 37,1 | | 27,0 | | 16,3 | 19,6 | 64,1 | 35,9 | 0,1562 | -0,0911 |
|  |  | *S. anomalus* | | Caucasian squirrel / 345 | | | | | 2,547 | | 35,3 | | 25,3 | | 17,5 | 21,9 | 60,6 | 39,4 | 0,164 | −0.113 |
|  |  | *S. vulgaris* | | Eurasian red squirrel / 1418 | | | | | 2,547 | | 35,8 | | 26,6 | | 16,8 | 20,8 | 62,4 | 37,6 | 0,147 | −0.106 |
| **D-loop** | | *D. nitedula* | | Forest dormouse / 754 | | | | | 1,140 | | 32,7 | | 29,1 | | 13,7 | 24,5 | 61,8 | 38,2 | 0,0581 | -0,2827 |
|  |  | *D. nitedula* | | Forest dormouse / 1878 | | | | | 1,136 | | 32,8 | | 28,9 | | 13,0 | 25,3 | 61,7 | 38,3 | 0,0641 | -0,3195 |
|  |  | *D. laniger* | | Woolly dormouse / 1899 | | | | | 1,146 | | 32,2 | | 20,8 | | 12,3 | 24,7 | 63,0 | 37,0 | 0,0221 | -0,3349 |
|  |  | *G. glis* | | Fat dormouse / 1832 | | | | | 1,158 | | 33,2 | | 31,1 | | 10,4 | 25,3 | 64,2 | 35,8 | 0,0322 | -0,4154 |
|  |  | *S. xanthoprymnus* | | Anatolian ground squirrel / 250 | | | | | 1,009 | | 31,1 | | 32,9 | | 12,1 | 23,9 | 64,0 | 36,0 | -0,0278 | -0,3278 |
|  |  | *S. citellus* | | European suslik / 336 | | | | | 1,000 | | 31,8 | | 32,5 | | 12,1 | 23,6 | 64,3 | 35,7 | -0,0108 | -0,3221 |
|  |  | *S. taurensis* | | Taurus ground squirrel / 339 | | | | | 1,001 | | 32,0 | | 32,6 | | 11,9 | 23,6 | 64,5 | 35,5 | -0,0092 | -0,3295 |
|  |  | *S. anomalus* | | Caucasian squirrel / 345 | | | | | 1,054 | | 29,5 | | 32,9 | | 12,3 | 25,3 | 62,4 | 37,6 | −0.055 | −0.348 |
|  |  | *S. vulgaris* | | Eurasian red squirrel / 1418 | | | | | 1,058 | | 30,1 | | 33,6 | | 11,8 | 24,5 | 63,7 | 36,3 | −0.056 | −0.349 |
