# Supplementary figures and images for: Mitogenomic Analysis of Glirids (Gliridae) and Squirrels (Sciuridae) From Türkiye: Evolutionary and Taxonomic Implications Within the Suborder Sciuromorpha
Source: Ecol Evol. 2025 Feb 12;15(2):e70956. doi: 10.1002/ece3.70956 (PMC11821457; doi:10.1002/ece3.70956)

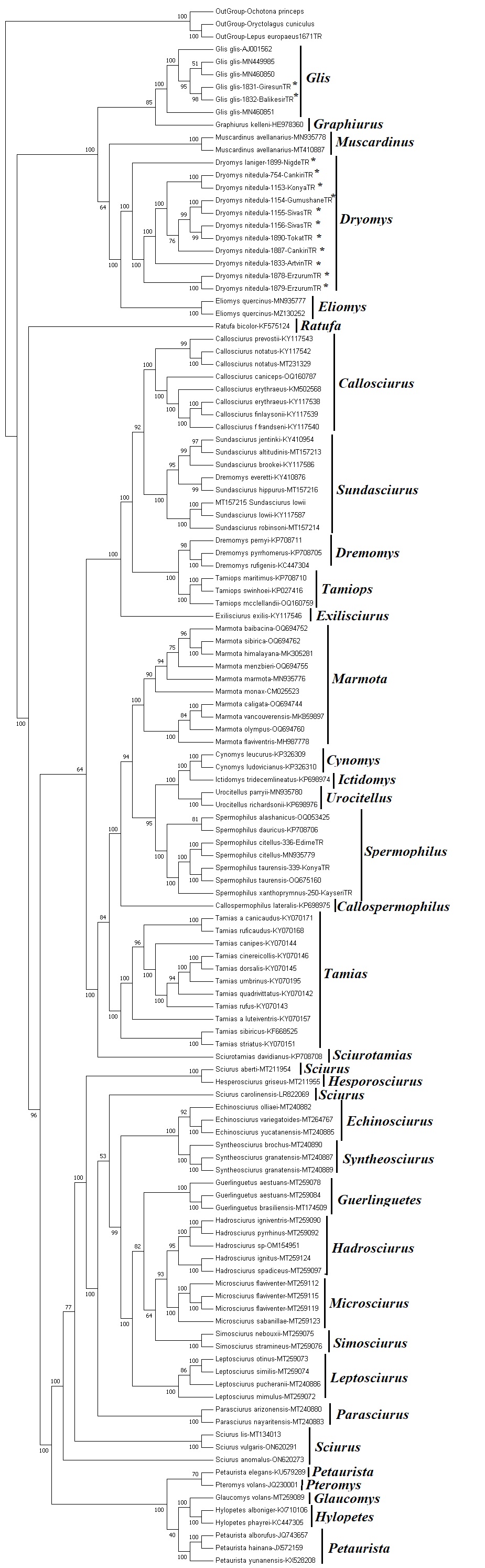

Supplement: Supplementary file 6 — File S6. ML phylogenetic tree reconstructed using mitogenomes for Sciuromorpha (Gliridae and Sciuridae) based on the GTR+G+I model and 1000 bootstrap replicates. [file ECE3-15-e70956-s005.jpg]

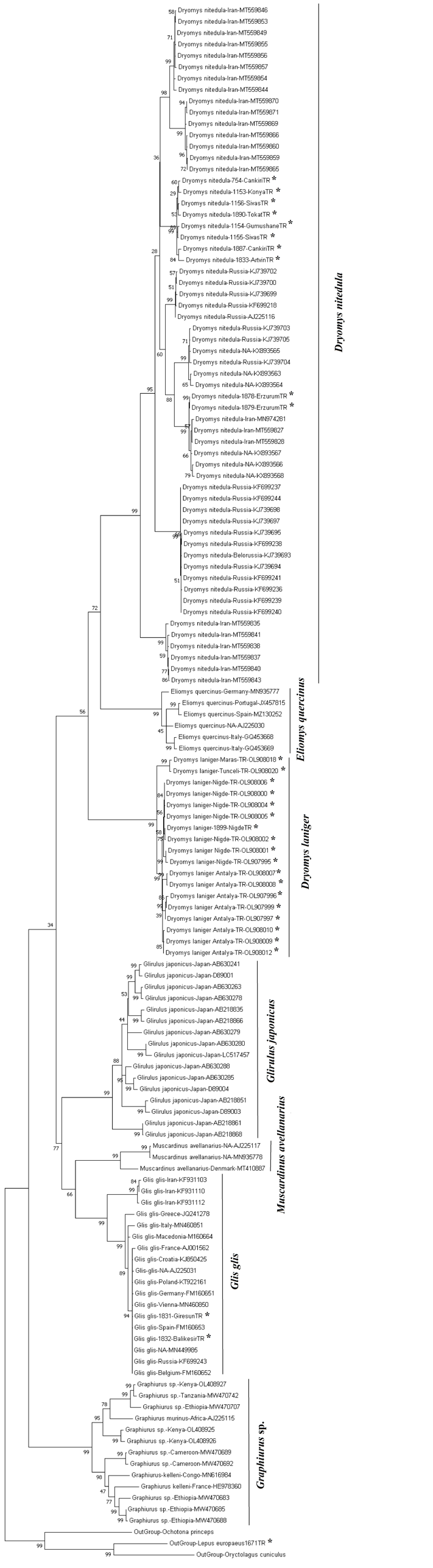

Supplement: Supplementary file 8 — File S8. ML phylogenetic tree reconstructed using mitochondrial CYTB sequences for Gliridae based on the GTR+G+I model and 10,000 bootstrap replicates. [file ECE3-15-e70956-s007.jpg]
