# Supplementary material for: Mitogenomic Analysis of Glirids (Gliridae) and Squirrels (Sciuridae) From Türkiye: Evolutionary and Taxonomic Implications Within the Suborder Sciuromorpha
Source: Ecol Evol. 2025 Feb 12;15(2):e70956. doi: 10.1002/ece3.70956 (PMC11821457; doi:10.1002/ece3.70956)
Supplement: Supplementary file 7 — File S7. Haplotypes of CYTB sequences obtained from the NCBI database and Türkiye, and summary statistics of sequence variation for Dryomys laniger and D. nitedula . [file ECE3-15-e70956-s002.docx]

**Supplement file 7.** Haplotypes of *CYTB* sequences obtained from the NCBI database and samples collected in Türkiye, and summary statistics of sequence variation, for *Dryomys laniger* and *D. nitedula*.

| ***Dryomys laniger*** | | | | |
| --- | --- | --- | --- | --- |
| **Haplotype name** | | **Freq.** | **Sequences used in phylogenetic analyses** | **The identic sequences and their locations** (not included in phylogenetic analyses) |
| **Hap_1** | | **1** | Dryomys_laniger-1899-NigdeTR |  |
| **Hap_2** | | **1** | Dryomys_laniger-Nigde-TR-OL908002 |  |
| **Hap_3** | | **2** | Dryomys_laniger-Nigde-TR-OL908004 | OL908003: Nigde, Turkiye |
| **Hap_4** | | **1** | Dryomys_laniger-Nigde-TR-OL908006 |  |
| **Hap_5** | | **1** | Dryomys_laniger-Nigde-TR-OL908005 |  |
| **Hap_6** | | **1** | Dryomys_laniger_Nigde-TR-OL908001 |  |
| **Hap_7** | | **1** | Dryomys_laniger-Nigde-TR-OL908000 |  |
| **Hap_8** | | **2** | Dryomys_laniger_Antalya-TR-OL908010 | OL908011: Antalya, Turkiye |
| **Hap_9** | | **1** | Dryomys_laniger_Antalya-TR-OL908012 |  |
| **Hap_10** | | **1** | Dryomys_laniger_Antalya-TR-OL908009 |  |
| **Hap_11** | | **1** | Dryomys_laniger_Antalya-TR-OL908007 |  |
| **Hap_12** | | **1** | Dryomys_laniger_Antalya-TR-OL908008 |  |
| **Hap_13** | | **2** | Dryomys_laniger_Antalya-TR-OL907997 | OL907998: Antalya, Turkiye |
| **Hap_14** | | **1** | Dryomys_laniger_Antalya-TR-OL907999 |  |
| **Hap_15** | | **1** | Dryomys_laniger_Antalya-TR-OL907996 |  |
| **Hap_16** | | **1** | Dryomys_laniger-Nigde-TR-OL907995 |  |
| **Hap_17** | | **6** | Dryomys_laniger-Maras-TR-OL908018 | OL908013: Maras, Turkiye  OL908014: Maras, Turkiye  OL908015: Maras, Turkiye  OL908016: Maras, Turkiye  OL908017: Maras, Turkiye |
| **Hap_18** | | **5** | Dryomys_laniger-Tunceli-TR-OL908020 | OL908019: Tunceli, Turkiye  OL908021: Tunceli, Turkiye  OL908022: Tunceli, Turkiye  OL908023: Tunceli, Turkiye |
|  | **Number of Haplotypes, h** | | | **18** |
|  | **Haplotype diversity, Hd** | | | **0.936** |
|  | **Variance of Haplotype diversity** | | | **0.00083** |
|  | **Standard Deviation of Haplotype diversity** | | | **0.029** |
|  | **Number of variable sites** | | | **130** |
|  | **Parsimony informative sites** | | | **105** |
|  | **Nucleotide diversity, Pi** | | | **0.03875** |
|  | **Fu's Fs statistic** | | | **4.741** |
|  | **Strobeck's S statistic** | | | **0.024** |
|  | **Tajima's D** | | | **0.97231** |
| ***Dryomys nitedula*** | | | | |
| **Haplotype name** | | **Freq.** | **Sequences used in phylogenetic analyses** | **The identic sequences and their locations** (not included in phylogenetic analyses) |
| **Hap_1** | | **1** | Dryomys_nitedula-1887-CankiriTR |  |
| **Hap_2** | | **1** | Dryomys_nitedula-1890-TokatTR |  |
| **Hap_3** | | **1** | Dryomys_nitedula-1156-SivasTR |  |
| **Hap_4** | | **1** | Dryomys_nitedula-1153-KonyaTR |  |
| **Hap_5** | | **1** | Dryomys_nitedula-754-CankiriTR |  |
| **Hap_6** | | **1** | Dryomys_nitedula-1154-GumushaneTR |  |
| **Hap_7** | | **1** | Dryomys_nitedula-1155-SivasTR |  |
| **Hap_8** | | **1** | Dryomys_nitedula-1833-ArtvinTR |  |
| **Hap_9** | | **1** | Dryomys_nitedula-Iran-MT559844 |  |
| **Hap_10** | | **1** | Dryomys_nitedula-Iran-MT559854 |  |
| **Hap_11** | | **5** | Dryomys_nitedula-Iran-MT559855 | MT559845: Iran  MT559847: Iran  MT559850: Iran  MT559852: Iran |
| **Hap_12** | | **3** | Dryomys_nitedula-Iran-MT559856 | MT559848: Iran  MT559851: Iran |
| **Hap_13** | | **1** | Dryomys_nitedula-Iran-MT559849 |  |
| **Hap_14** | | **1** | Dryomys_nitedula-Iran-MT559846 |  |
| **Hap_15** | | **1** | Dryomys_nitedula-Iran-MT559853 |  |
| **Hap_16** | | **1** | Dryomys_nitedula-Iran-MT559857 |  |
| **Hap_17** | | **4** | Dryomys_nitedula-Iran-MT559866 | MT559858: Iran  MT559861: Iran  MT559864: Iran |
| **Hap_18** | | **2** | Dryomys_nitedula-Iran-MT559860 | MT559868: Iran |
| **Hap_19** | | **3** | Dryomys_nitedula-Iran-MT559865 | MT559862: Iran  MT559863: Iran |
| **Hap_20** | | **2** | Dryomys_nitedula-Iran-MT559859 | MT559867: Iran |
| **Hap_21** | | **1** | Dryomys_nitedula-Iran-MT559869 |  |
| **Hap_22** | | **1** | Dryomys_nitedula-Iran-MT559870 |  |
| **Hap_23** | | **1** | Dryomys_nitedula-Iran-MT559871 |  |
| **Hap_24** | | **1** | Dryomys_nitedula-Russia-KJ739702 |  |
| **Hap_25** | | **2** | Dryomys_nitedula-Russia-KJ739700 | KF699219: Russia |
| **Hap_26** | | **1** | Dryomys_nitedula-Russia-AJ225116 |  |
| **Hap_27** | | **1** | Dryomys_nitedula-Russia-KJ739699 |  |
| **Hap_28** | | **2** | Dryomys_nitedula-Russia-KF699218 | KJ739701: Russia |
| **Hap_29** | | **2** | Dryomys_nitedula-1879-ErzurumTR | Dryomys_nitedula-1878-Erzurum, Turkiye |
| **Hap_30** | | **1** | Dryomys_nitedula-NA-KX893566 |  |
| **Hap_31** | | **1** | Dryomys_nitedula-NA-KX893568 |  |
| **Hap_32** | | **1** | Dryomys_nitedula-NA-KX893567 |  |
| **Hap_33** | | **3** | Dryomys_nitedula-Iran-MT559827 | MT559829: Iran  MT559831: Iran |
| **Hap_34** | | **3** | Dryomys_nitedula-Iran-MT559828 | MT559830: Iran  MT559832: Iran |
| **Hap_35** | | **1** | Dryomys_nitedula-Iran-MN974281 |  |
| **Hap_36** | | **1** | Dryomys_nitedula-Russia-KJ739704 |  |
| **Hap_37** | | **1** | Dryomys_nitedula-Russia-KJ739705 |  |
| **Hap_38** | | **1** | Dryomys_nitedula-Russia-KJ739703 |  |
| **Hap_39** | | **1** | Dryomys_nitedula-NA-KX893565 |  |
| **Hap_40** | | **1** | Dryomys_nitedula-NA-KX893563 |  |
| **Hap_41** | | **1** | Dryomys_nitedula-NA-KX893564 |  |
| **Hap_42** | | **1** | Dryomys_nitedula-Belorussia-KJ739693 |  |
| **Hap_43** | | **3** | Dryomys_nitedula-Russia-KF699241 | KX893562: N/A  KJ739696: Russia |
| **Hap_44** | | **1** | Dryomys_nitedula-Russia-KJ739694 |  |
| **Hap_45** | | **1** | Dryomys_nitedula-Russia-KJ739695 |  |
| **Hap_46** | | **1** | Dryomys_nitedula-Russia-KJ739697 |  |
| **Hap_47** | | **1** | Dryomys_nitedula-Russia-KJ739698 |  |
| **Hap_48** | | **1** | Dryomys_nitedula-Russia-KF699239 |  |
| **Hap_49** | | **1** | Dryomys_nitedula-Russia-KF699240 |  |
| **Hap_50** | | **1** | Dryomys_nitedula-Russia-KF699244 |  |
| **Hap_51** | | **1** | Dryomys_nitedula-Russia-KF699237 |  |
| **Hap_52** | | **1** | Dryomys_nitedula-Russia-KF699238 |  |
| **Hap_53** | | **1** | Dryomys_nitedula-Russia-KF699236 |  |
| **Hap_54** | | **3** | Dryomys_nitedula-Iran-MT559835 | MT559833: Iran  MT559834: Iran |
| **Hap_55** | | **3** | Dryomys_nitedula-Iran-MT559840 | MT559836: Iran  MT559839: Iran |
| **Hap_56** | | **1** | Dryomys_nitedula-Iran-MT559837 |  |
| **Hap_57** | | **1** | Dryomys_nitedula-Iran-MT559843 |  |
| **Hap_58** | | **2** | Dryomys_nitedula-Iran-MT559838 | MT559842: Iran |
| **Hap_59** | | **1** | Dryomys_nitedula-Iran-MT559841 |  |
|  | |  |  |  |
|  | **Number of haplotypes, h** | | | **59** |
|  | **Haplotype diversity, Hd** | | | **0.9850** |
|  | **Variance of Haplotype diversity** | | | **0.00003** |
|  | **Standard Deviation of Haplotype diversity** | | | **0.005** |
|  | **Number of variable sites** | | | **309** |
|  | **Parsimony informative sites** | | | **280** |
|  | **Nucleotide diversity, Pi** | | | **0.08067** |
|  | **Fu's Fs statistic** | | | **1.452** |
|  | **Strobeck's S statistic** | | | **0.261** |
|  | **Tajima's D** | | | **1.04798** |
